# An Oxidative Stress-Related Gene Pair (*CCNB1*/*PKD1*), Competitive Endogenous RNAs, and Immune-Infiltration Patterns Potentially Regulate Intervertebral Disc Degeneration Development

**DOI:** 10.3389/fimmu.2021.765382

**Published:** 2021-11-09

**Authors:** Shuai Cao, Hao Liu, Jiaxin Fan, Kai Yang, Baohui Yang, Jie Wang, Jie Li, Liesu Meng, Haopeng Li

**Affiliations:** ^1^ Department of Orthopaedics, The Second Affiliated Hospital of Xi’an Jiaotong University, Xi’an, China; ^2^ Department of Neurology, The Second Affiliated Hospital of Xi’an Jiaotong University, Xi’an, China; ^3^ Department of Orthopaedic Surgery, Guangdong Provincial Key Laboratory of Orthopedics and Traumatology, First Affiliated Hospital of Sun Yat-sen University, Guangzhou, China; ^4^ National & Local Joint Engineering Research Center of Biodiagnostics and Biotherapy, The Second Affiliated Hospital of Xi’an Jiaotong University, Xi’an, China; ^5^ Key Laboratory of Environment and Genes Related to Diseases, Ministry of Education of China, Xi’an, China

**Keywords:** intervertebral disc degeneration, oxidative stress, gene pair, immune infiltration, competitive endogenous RNA, differential expression, cyclin B1, protein kinase D1

## Abstract

Oxidative stress (OS) irreversibly affects the pathogenesis of intervertebral disc degeneration (IDD). Certain non-coding RNAs act as competitive endogenous RNAs (ceRNAs) that regulate IDD progression. Analyzing the signatures of oxidative stress-related gene (OSRG) pairs and regulatory ceRNA mechanisms and immune-infiltration patterns associated with IDD may enable researchers to distinguish IDD and reveal the underlying mechanisms. In this study, OSRGs were downloaded and identified using the Gene Expression Omnibus database. Functional-enrichment analysis revealed the involvement of oxidative stress-related pathways and processes, and a ceRNA network was generated. Differentially expressed oxidative stress-related genes (De-OSRGs) were used to construct De-OSRG pairs, which were screened, and candidate De-OSRG pairs were identified. Immune cell-related gene pairs were selected *via* immune-infiltration analysis. A potential long non-coding RNA–microRNA–mRNA axis was determined, and clinical values were assessed. Eighteen De-OSRGs were identified that were primarily related to intricate signal-transduction pathways, apoptosis-related biological processes, and multiple kinase-related molecular functions. A ceRNA network consisting of 653 long non-coding RNA–microRNA links and 42 mRNA–miRNA links was constructed. Three candidate De-OSRG pairs were screened out from 13 De-OSRG pairs. The abundances of resting memory CD4^+^ T cells, resting dendritic cells, and CD8^+^ T cells differed between the control and IDD groups. CD8^+^ T cell infiltration correlated negatively with cyclin B1 (*CCNB1*) expression and positively with protein kinase D1 (*PKD1*) expression. *CCNB1*–*PKD1* was the only pair that was differentially expressed in IDD, was correlated with CD8^+^ T cells, and displayed better predictive accuracy compared to individual genes. The *PKD1*–*miR-20b-5p*–*AP000797* and *CCNB1*–*miR-212-3p*–*AC079834* axes may regulate IDD. Our findings indicate that the OSRG pair *CCNB1*–*PKD1*, which regulates oxidative stress during IDD development, is a robust signature for identifying IDD. This OSRG pair and increased infiltration of CD8^+^ T cells, which play important roles in IDD, were functionally associated. Thus, the OSRG pair *CCNB1*–*PKD1* is promising target for treating IDD.

## Introduction

Intervertebral disc degeneration (IDD) is a major factor inducing chronic lower back pain. Moreover, IDD frequently causes injury to the spinal cord and related nerves, which has important clinical implications when the contours change or contents leak (1). Severe health-related disabilities and enormous economic losses caused by IDD have drawn global attention (2, 3). The intervertebral disc, which lacks vasculature, comprises the interior nucleus pulposus (NP), outer annulus fibrosus, and thin cartilaginous endplates (4). Multiple risk factors, such as heredity, age, smoking, circadian rhythms, and high mechanical compression, contribute to the nutrient insufficiency and imbalanced acid–base homeostasis observed in NP cells (2, 5). Because of the early initiation of degenerative changes and absence of apparent symptoms, most patients with IDD are identified at an advanced stage, invasive surgery as the only treatment option, which has a poor prognosis. Thus, novel approaches that enable rapid detection of IDD pathogenesis and early diagnoses are urgently needed.

According to prevailing concepts, IDD pathogenesis centers on autophagy, apoptosis, inflammation, and metabolism (6–9). However, increasing evidence has shown that oxidative stress, caused by excessive accumulation of reactive oxygen species (ROS), plays a crucial role in driving IDD initiation and progression (10, 11). ROS, such as superoxide anions, hydrogen peroxide, and singlet oxygen, are byproducts of cellular oxidative metabolism (12). The physiological equilibrium is disturbed once the rate of ROS production exceeds that of its elimination within NP cells. Subsequently, ROS-driven oxidative stress causes time-dependent damage to DNA and proteins, which is compounded by coexisting cellular damage. The levels of some oxidation products of ROS or substances that participate in ROS metabolism (such as malondialdehyde, peroxynitrite, and glutathione) are elevated in patients with IDD (13, 14). Nevertheless, the precise underlying mechanisms and potential markers of oxidative stress-related genes (OSRGs) in IDD remain unclear.

In recent decades, non-coding RNAs, including microRNAs (miRNAs) and long non-coding RNAs (lncRNAs), have gained attention. Various non-coding RNAs have been identified as effective prognostic or diagnostic molecular signatures, particularly in the field of oncology (15, 16). Over 80% of transcripts in the human genome are not translated into proteins and remain as non-coding sequences (17). Among these, transcripts longer than 200 nucleotides are recognized as lncRNAs (18), whereas those with 18–22 nucleotides are classified as miRNAs (19). With the rapid evolution of sequencing technologies, a growing number of lncRNAs, such as *ANPODRT* (20), *FAM83H-AS1* (21), and *TRPC7-AS1* (22), has been considered as essential regulators of gene expression in IDD at the transcriptional, post-transcriptional, and epigenetic levels. According to the competing-endogenous RNA (ceRNA) hypothesis, regulatory lncRNAs interact with miRNAs by serving as decoys for specific miRNAs, resulting in changes in the expression of target mRNAs (23, 24). To date, relatively few studies have focused on oxidative stress-associated ceRNA networks. An in-depth understanding of ceRNA crosstalk may reveal the mechanisms underlying IDD pathogenesis.

Additionally, some components of NP cells are gradually exposed to the body’s immune system during IDD development, thereby triggering a series of auto-immune responses (25). The activities of various infiltrating immune cells, such as macrophages (26) and CD8^+^ T cells (27), which are present in this inflammatory microenvironment, contribute to the exacerbation of IDD. The mechanism(s) underlying oxidative stress and its association with immune privilege in human IDD remain unclear. Therefore, studies are needed to clarify the underlying immunological mechanisms and develop novel immunotherapeutic targets for IDD.

In this study, we investigated differentially expressed oxidative stress-related genes (De-OSRGs) using publicly available Gene Expression Omnibus (GEO) microarray sets and OSRGs associated with IDD. Simple batch-correction methods often do not fully eliminate batch effects. Hence, we constructed De-OSRG pairs by merging different datasets and establishing and annotating a ceRNA network based on the De-OSRGs. Moreover, machine-learning algorithms were used to screen for candidate De-OSRG pairs. Their associations with immune-infiltration features were further investigated to determine the optimal oxidative stress-related signature. Based on the results, a receiver operating characteristic (ROC) curve, which evaluates distinguishing performers in IDD, was developed, and potential regulatory mechanisms were identified.

## Materials and Methods

### Data Acquisition and Preprocessing

**Figure 1:**
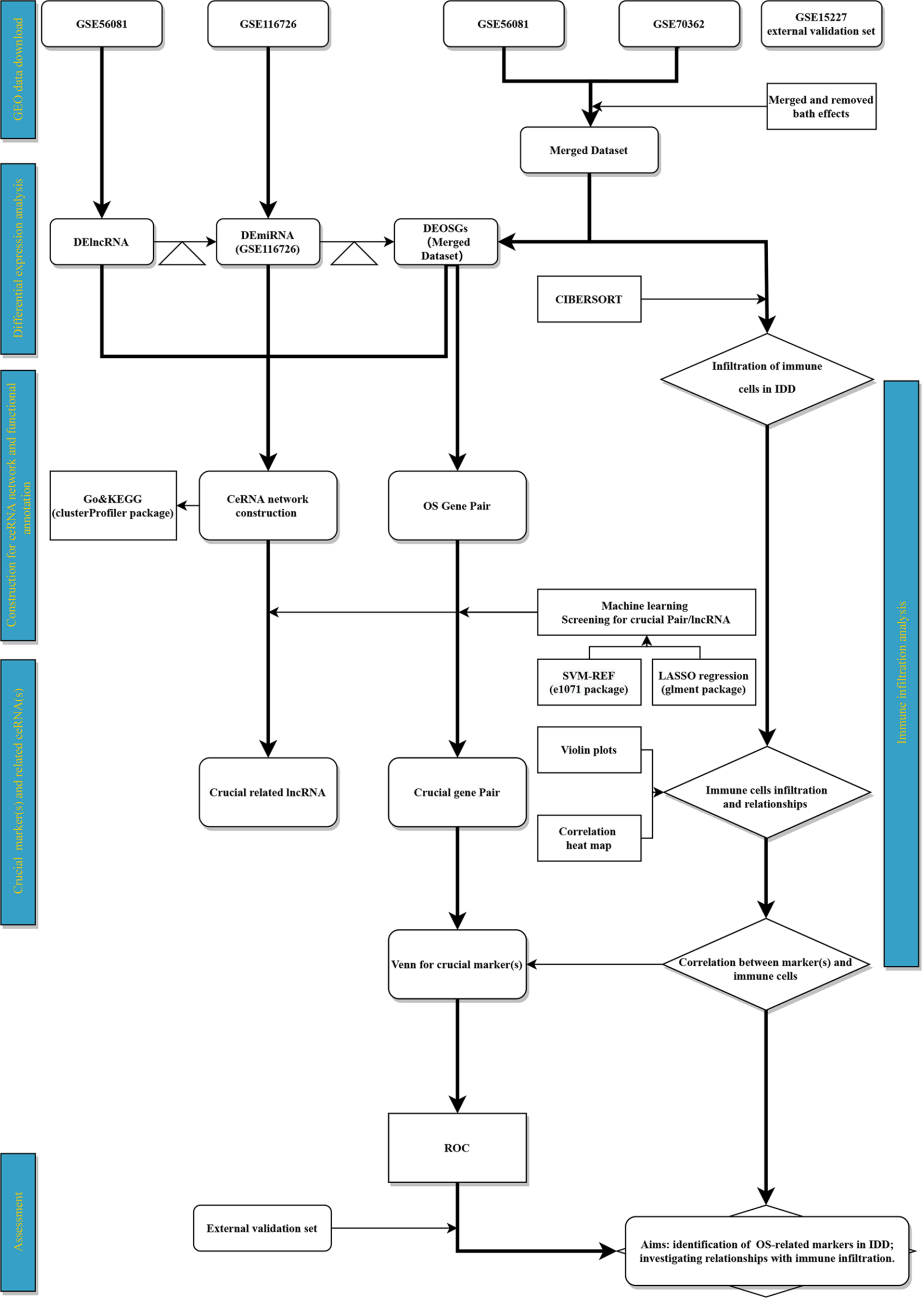
Flowchart. Triangle represents target‐gene prediction (first triangle, lncRNA target prediction; second triangle, miRNA target prediction).

The gene-expression profiles of IDD, including GSE116726 (miRNA set), GSE56081 (lncRNA and mRNA sets), GSE70362 (mRNA set), and GSE15227 (mRNA and external validation sets), were retrieved (**Supplementary Table S1**) and collected using the GEO database (URL: **Supplementary Table S2**). The GSE116726 set consists of three controls (none female and three males) and three patients with IDD (none female and three males) from China, whose average age was 56 years. Five controls (one female and four males; average age 40.8 years) and five patients with IDD (two female and three males; average age 36.8 years) from China comprised the GSE56081 set. Fourteen controls (seven female and seven males; average age 48.9 years) and ten patients (three female and seven males; average age 74.8 years) with IDD from Ireland made up the GSE70362 set. The GSE15227 set consisted of twelve controls and three patients with IDD from the USA. However, information of sex and age in the GSE152227 set was not reported. The details of the clinical information in each data set are shown in **Supplementary Table S3**. All 1399 OSRGs obtained from the GeneCards database (URL: **Supplementary Table S2**) are listed in **Supplementary Table S4**. Consistent with the Thompson grading system (28), NP samples were divided into two categories: a control group that comprised grades I–III and IDD group that comprised grades IV–V. We preprocessed the raw data using the following methods: i) merging GSE56081, GSE70362, and OSRGs into a dataset (merged dataset) to obtain a sample size that was sufficient for mRNA analysis; and (ii) performing batch normalization using the “sva” package of R to maintain the homogeneity of these sets. The flowchart and details of data processing are shown in **Figure 1**. Public access and publication guidelines approved by the GEO database were strictly followed when obtaining public data. Approval from the ethics committee of the Second Affiliated Hospital of Xi’an Jiaotong University was not required for this study.

### Identifying De-OSRGs

First, we performed expression validation using the “limma” package in R software to identify differentially expressed mRNAs, miRNAs, and lncRNAs (DEmRNAs, DEmiRNAs, and DElncRNAs, respectively) between the IDD and control groups in the expanded datasets (GSE116726 and GSE56081, respectively). To visualize the differences, volcano maps and clustering heat maps were established using the “ggplot2” and “pheatmap” packages in R. To ensure the reliability of the data, we performed online database predictions for the DElncRNAs to obtain target mRNAs. Briefly, miRDB, miRTarBase, and TargetScan (URL: **Supplementary Table S2**) were used to predict miRNA–mRNA interactions, and miRcode (URL: **Supplementary Table S2**) was utilized to predict lncRNA–miRNA interactions. Genes that overlapped between the DEmRNAs and target mRNAs were regarded as De-OSRGs. A P value of <0.05 and fold-change in expression of >1.5 were used as the screening threshold.

In addition, a ceRNA network was constructed with the De-OSRGs and their target DEmiRNAs, along with target DElncRNAs, and visualized using Cytoscape software (v3.8.0) (29).

### Functional Annotation of the ceRNA Network

To systematically annotate the potential functions and pathways of the De-OSRGs, Kyoto Encyclopedia of Genes and Genomes (KEGG) and Gene Ontology (GO) analyses were conducted using the “enrichplot”, “clusterProfile”, “ggplot2”, and “org.Hs.eg.db” packages in R (30). GO terms, consisting of molecular functions (MFs) and biological processes (BPs), were used. Statistical significance was set at P < 0.05.

### Computation of De-OSRG Pairs

Using the identified De-OSRGs, we calculated De-OSRG pairs according to the computation rules described by Zhang et al. (15). Briefly, if Expr_OSRGa_ < Expr_OSRGb_, then OSRGP = 1; otherwise, OSRGP = 0, where Expr_OSRGa_ denotes the expression level of OSRG a, and Expr_OSRGb_ denotes the expression level of OSRG b. Thus, each OSRG pair was considered to be comprised of 0s and 1s. Following these pair-comparison rules, the numerical OSRG pair levels of each dataset were computed separately.

### Screening for Crucial De-OSRG Pairs and Candidate Signatures

The least-absolute shrinkage and selection operator (LASSO) regression and support vector machine (SVM) machine-learning algorithms can be used to reduce the number of feature variables (31). The former algorithm preserves a variable by finding the best penalty parameter, λ, when the classification error is minimal, whereas the latter algorithm searches for the best optimal hyperplane that classifies different groups (in our case, IDD and control patients). We integrated overlapping gene pairs, which were screened *via* LASSO regression and SVM, by considering them as candidate OSRG pair signatures for further analysis.

### Estimating Immune-Infiltration Patterns

CIBERSORT, a computer analysis tool, is widely used to evaluate the abundance of immune cells and assess the proportions of various immune cells using RNA-sequencing-based expression values (32). Herein, the candidate signatures were subjected to immune-infiltration analysis using the “CIBERSORT”, “parallel”, “preprocessCore”, and “e1071” packages in R. Based on previous findings (31), we selected 22 immune cell subsets for analysis (32). A histogram was used to visualize the distributions of infiltrating immune cells in each subject. A correlation heatmap was drawn to reveal correlations between infiltrating immune cells using the “corrplot” package in R. Furthermore, a violin diagram was generated to visualize differences between the IDD and normal groups. The filtering threshold was set at P < 0.05.

### Correlation Analysis Between Infiltrating Immune Cells and Candidate OSRGs

Spearman correlation analysis between infiltrating immune cells and candidate OSRGs was performed using the “limma” package in R and visualized using the “ggplot2”, “tidyverse”, and “ggsci” packages in R.

### Screening for Key ceRNAs With Crucial De-OSRG Pairs

We screened ceRNAs with crucial gene pairs *via* LASSO regression and SVM. These ceRNAs were further analyzed.

### Performance Evaluations

De-OSRG pairs-based ROC curve analysis was performed using the “pROC” package in R to distinguish IDD (33). We merged GSE56081 with GSE70362 as a training set and used GSE15227 as a validation set. The ROC curve, based on logistic regression, was evaluated for its efficacy in identifying IDD. In addition, we compared the areas under the receiver operating curves (AUCs) of separate genes and gene pairs, as well as the associated specificities, sensitivities, and 95% confidence intervals (CIs). The AUC was calculated separately to evaluate the performance/distinguishing ability of separate genes and gene pairs. Moreover, the external validation dataset was used to assess the De-OSRG pairs that distinguished IDD *via* ROCs.

### Statistical Analysis

All statistical analyses were performed using R software (v3.6.2, R Core Team, The R Project for Statistical Computing, Vienna, Austria) and MedCalc statistical software (v19.0.4, MedCalc, Inc., Oostende, Belgium). GraphPad Prism (v8.0.1, GraphPad, Inc., La Jolla, CA, USA), Cytoscape (v3.8.0), and R software were used to generate graphics. All R packages used for analysis are listed in **Supplementary Table S5**. Upregulated expression was defined as a log_2_ fold-change of >0, whereas downregulated expression was defined as a log_2_ fold-change of <0. Statistical significance for each test was set at P < 0.05.

## Results

### Identification of 18 De-OSRGs and Construction of a ceRNA Network

**Figure 2:**
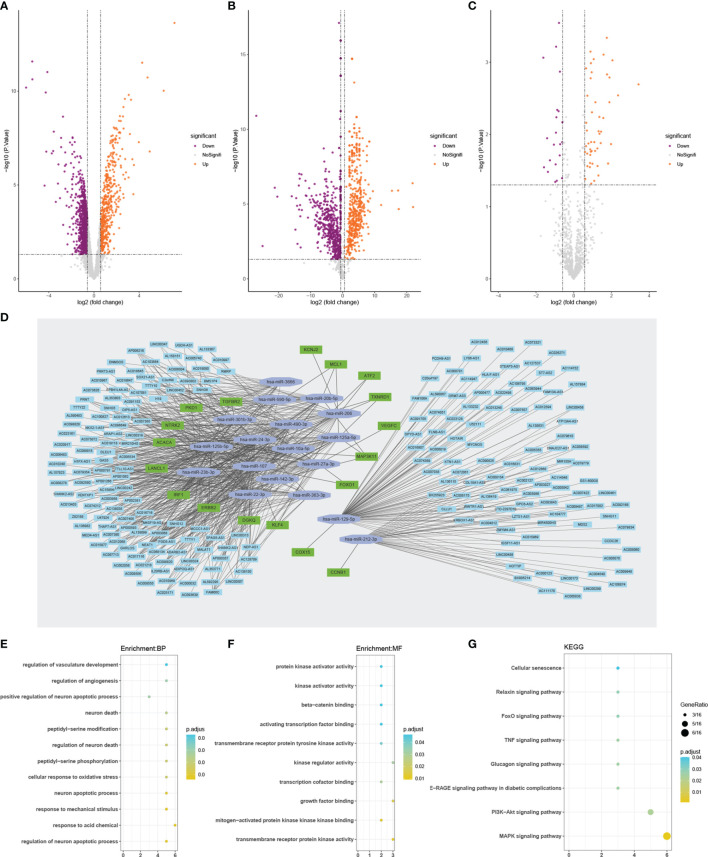
Volcano plots of DEGs **(A–C)**. **(A)** Volcano plot of De–lncRNAs. **(B)** Volcano plot of De-miRNAs. **(C)** Volcano plot of De-mRNAs. Orange represents up-regulated differentially expressed genes, while purple represents down-regulated differentially expressed genes. The ceRNA network analyses of 18 DEOSRGs, and their function annotation by GO and KEGG **(D–G)**. **(D)** The ceRNA network analysis graph. GO enrichment analysis of 18 DEOSRGs **(E, F)**. **(E)** BP, biological process; **(F)** MF, molecular function. **(G)** KEGG pathway enrichment analysis of 18 DEOSRGs. De-lncRNAs, differentially expressed long noncoding RNAs; De–miRNAs, differentially expressed microRNAs; De-mRNAs, differentially expressed mRNAs; De–OSRGs, differentially expressed oxidative stress-related genes. GO, Gene Ontology; KEGG, Kyoto Encyclopedia of Genes and Genomes.

We identified 1428 DElncRNAs and 1176 DEmiRNAs using GSE56081 and GSE116726, respectively (**Figures 2A, B**). In addition, 69 DEmRNAs were extracted from the expanded dataset (**Figure 2C**). Based on online database predictions, 207 target miRNAs were predicted for 1428 DElncRNAs. Furthermore, 18 target miRNAs were obtained from miRNAs that overlapped between the 1176 DEmiRNAs and 207 miRNAs predicted by DElncRNAs. Eighteen De-OSRGs were associated with 69 DEmRNAs from the expanded dataset, along with 8612 predicted target mRNAs for miRNAs. Among these were 16 and 2 up- and downregulated De-OSRGs, respectively. The ceRNA network was comprised of 18 De-OSRGs, 211 target DElncRNAs, and 18 target DEmiRNAs (**Figure 2D**).

### Functional Annotation

GO annotation indicated that the 18 De-OSRGs mainly participated in oxidative stress-associated and apoptosis-related BPs, such as cellular response to oxidative stress and regulation of neuronal apoptotic processes (**Figure 2E** and **Supplementary Table S6**). MF annotation showed that the MFs were primarily associated with kinase-related functions, including mitogen-activated protein kinase (MAPK) binding, kinase-regulator activity, transmembrane receptor-tyrosine kinase activity, and protein kinase-activator activity (**Figure 2F** and **Supplementary Table S6**). Furthermore, Kyoto Encyclopedia of Genes and Genomes enrichment analysis suggested that the 18 De-OSRGs were principally involved in the MAPK signaling pathway, phosphoinositide 3-kinase-protein kinase B signaling pathway, relaxin signaling pathway, FoxO signaling pathway, and cellular senescence, indicating that these genes play important roles in oxidative stress (**Figure 2G** and **Supplementary Table S7**).

### Screening for Candidate Signatures of De-OSRG Pairs

**Figure 3:**
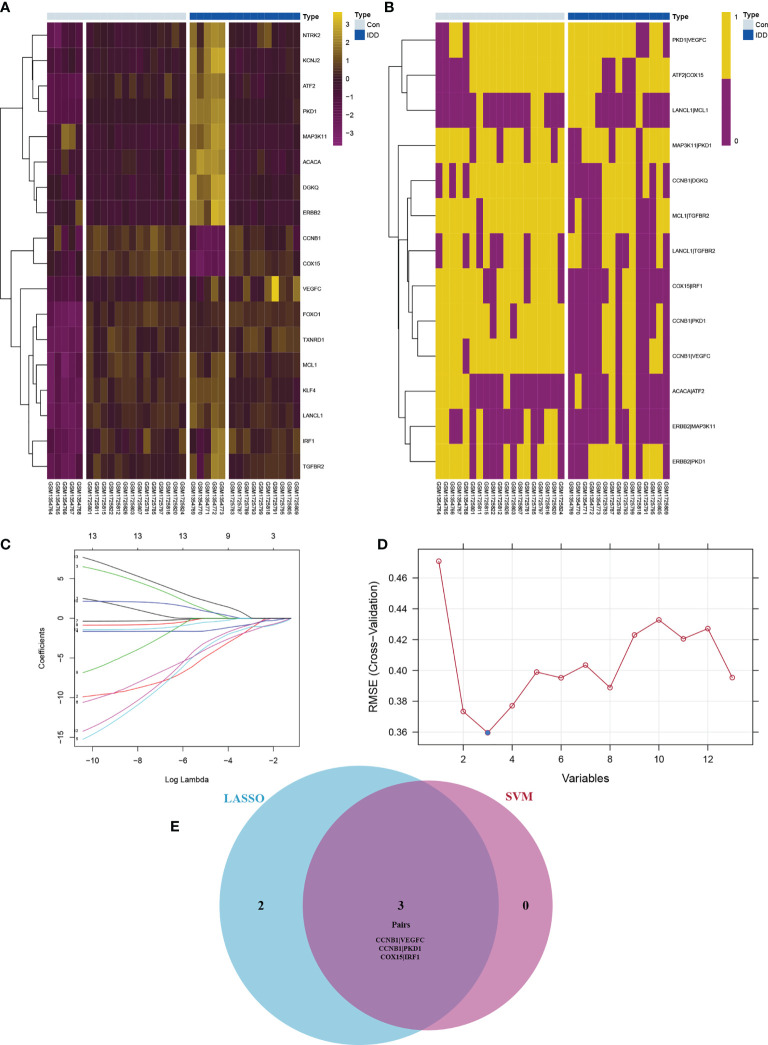
The expression profile for De-OSRGs **(A)** and De-OSRGPs **(B)**. The difference between OSRGs heat map and OSRGPs heat map **(A, B)**: although the batch correction is used, the gap between different datasets can be clearly shown in panel **(A)** The screening process of key OSRGPs for IDD. **(C)** LASSO regression to screen for candidate signatures. **(D)** SVM to screen for candidate signatures. **(E)** Venn diagram demonstrating the intersection of candidate signatures screened by LASSO regression and SVM. OSRGs, oxidative stress-related genes; OSRGPs, oxidative stress-related gene pairs; IDD, intervertebral disc degeneration; LASSO, least absolute shrinkage and selection operator; SVM, support vector machine.

Although batch normalization was performed, the expression heatmap of the 18 De-OSRGs showed gaps within the groups (**Figure 3A**). Hence, of the 18 De-OSRGs, 13 were established using filter values of 0.1–0.9 (**Figure 3B**). LASSO regression analysis (seed = 14) was used to select five gene pairs (**Figure 3C**). SVM analysis identified three gene pairs (seed = 14; **Figure 3D**) that intersected with the LASSO regression results (**Figure 3E**). We considered these three gene pairs, which included the following five OSRGs: cyclin B1 (CCNB1), protein kinase D1 (PKD1), cytochrome c oxidase assembly homolog 15, vascular endothelial growth factor C, and interferon regulatory factor 1, as candidate IDD signatures for further immune-infiltration analysis.

### Immune Cell Infiltration

**Figure 4:**
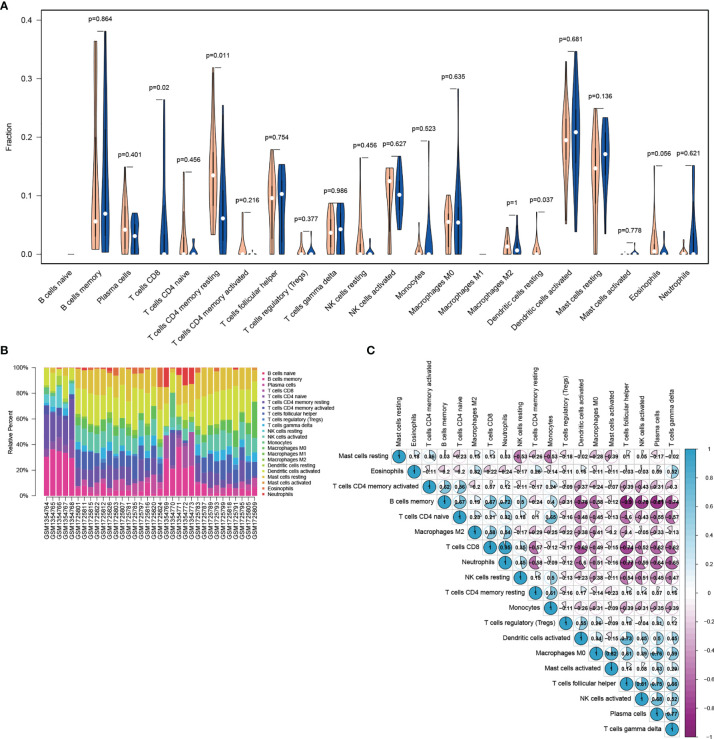
Visualization of immune cell infiltration analysis. **(A)** Violin diagram of the abundance of infiltration by 22 immune cell subsets between IDD and control groups. Blue and orange colors represent IDD and control groups, respectively. **(B)** Histogram of the distribution of infiltrating immune cells in each individual. Columns represent IDD individuals. **(C)** Correlation heatmap of 22 immune cell subsets. Blue represents positive correlation, whereas purple represents negative correlation. The color and number in each circle indicate the correlation index between the two immune cell subsets. IDD, intervertebral disc degeneration.

The violin diagram and histogram of immune cell infiltration clearly revealed abundant immune cells in IDD. The violin diagram indicated that resting memory CD4^+^ T cells and resting dendritic cells showed less infiltration in the IDD group, whereas CD8^+^ T cells showed more infiltration (**Figures 4A, B**). The correlation heatmap related to cell-type abundances indicated significant negative correlations between CD8^+^ T cells and resting memory CD4^+^ T cells and activated dendritic cells, and a positive correlation between CD8^+^ T cells and neutrophils. Resting memory CD4^+^ T cells showed a significant positive correlation with monocytes. Follicular helper T cells correlated positively with activated NK cells. Memory B cells correlated negatively with follicular helper T cells and plasma cells (**Figure 4C**). Immune infiltration analysis is predictive of specific infiltrating immune cell based on gene expression signatures.

### Correlation Analysis Between Infiltrating Immune Cells and Candidate De-OSRG Pairs

**Figure 5:**
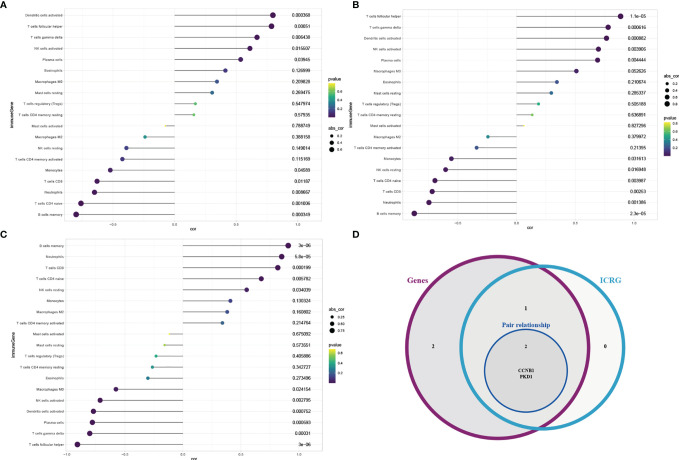
Correlation analysis between infiltrating immune cell subtypes with the candidate De-OSRGs. Spearman correlation analysis between immune cell subtypes and *CCNB1* **(A)** /*PKD1* **(B)** /*COX15* **(C)**. Venn diagram **(D)** showing the key gene pair shared by the immune cell-related genes (ICRG) and candidate De-OSRGs.

To select optimal OSRG pairs and clarify the associations between immune cell infiltration and OSRGs, we performed correlation analyses between infiltrating immune cells and the three candidate De-OSRG pairs. Correlation analysis showed that three of the five candidate De-OSRG pairs were prominently linked with various infiltrating immune cells (**Figures 5A–C**). Furthermore, significant differences were observed only between the numbers of CD8^+^ T cells, resting memory CD4^+^ T cells, and resting dendritic cells in the IDD and control groups. Interestingly, only the *CCNB1*–*PKD1* pair correlated with CD8^+^ T cells simultaneously. Specifically, *CCNB1* correlated negatively with CD8^+^ T cells (r = -0.630, P = 0.012; **Figure 5A**), and *PKD1* correlated positively with CD8^+^ T cells (r = 0.817, P < 0.001; **Figure 5B**). Therefore, we focused on the *CCNB1*–*PKD1* pair in further analysis (**Figure 5D**). Correlation analysis between infiltrating immune cell and candidate De-OSRG pairs was based on the gene expression signatures.

### Exploring Potential Regulatory Axes for the CCNB1–PKD1 Pair

**Figure 6:**
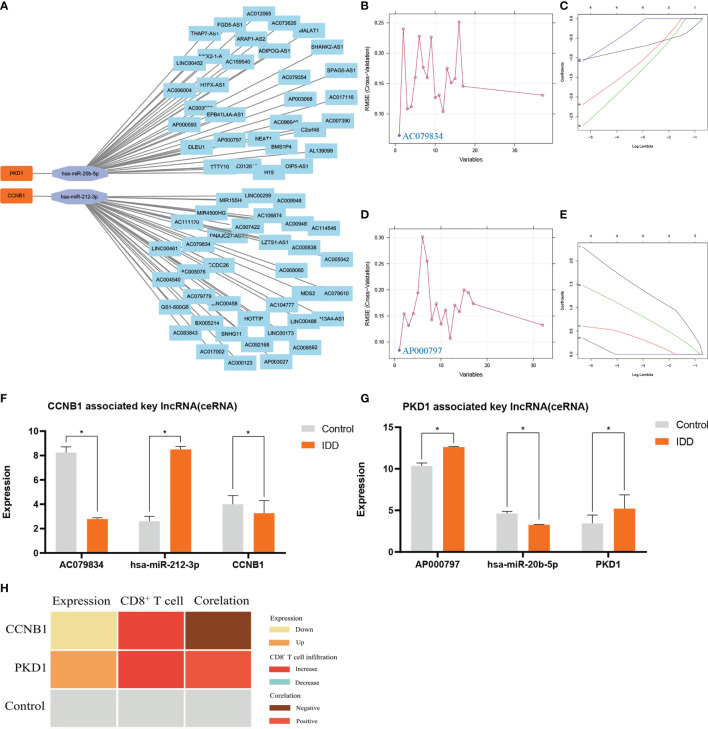
|The 69lncRNAs–2miRNAs–2mRNAs (*CCNB1*/*PKD1* pair) ceRNA co-expression network was constructed **(A)** and was used to screen the key ceRNAs (lncRNAs) by SVM **(B, D)** and Lasso **(C, E)**. Panels **(B, C)**, venn result for SVM **(B)** and LASSO **(C)** is *AC079834*. LncRNA *AC079834* was selected in SVM and LASSO, and was used for building axis (*CCNB1–miR-212-3p–AC079834*). Panels **(D, E)**, venn result for SVM **(D)** and LASSO **(E)** is *AP000797*. LncRNA *AP000797* was selected in SVM and LASSO, and was used for building axis (*PKD1–miR-20b-5p–AP000797*). Parameters and results in detail are shown in **Supplementary Tables S9, S10**.The expression profile for *CCNB1*–*miR-212-3p–AC079834* axis **(F)** and *PKD1*–*miR-20b-5p–AP000797* axis **(G)**. The low expression of *CCNB1* is negatively correlated with increased CD8^+^ T cells infiltration; the high expression of *PKD1* is positively correlated with increased CD8^+^ T cells infiltration **(H)**. * represents p < 0.05.

Focusing on the *CCNB1*–*PKD1*–ceRNA network axis (**Figure 6A**) evaluated using LASSO regression (seed = 25) and SVM analyses, key lncRNAs associated with these genes were revealed (seed = 25) (**Figures 6B–E**, respectively): lncRNA *AC079834* for *CCNB1* and lncRNA *AP000797* for *PKD1*. The parameters and results for screening the key lncRNA (ceRNA), which competes with *CCNB1* to bind miR-212-3p, are shown in **Supplementary Table S8**. The parameters and results for screening the key lncRNA (ceRNA), which competes with *PKD1* to bind miR-20b-5p, are shown in **Supplementary Table S9**. The *CCNB1*–*miR-212-3p*–*AC079834* and *PKD1*–*miR-20b-5p*–*AP000797* axes were identified as potential regulatory ceRNA axes. These findings indicate that the *CCNB1*–*PKD1* pair modulates oxidative stress through these axes during IDD initiation and development (**Figures 6F, G**). In summary, low *CCNB1* expression and high *PKD1* expression correlated negatively and positively, respectively, with increased CD8^+^ T cell infiltration (**Figure 6H**).

### Potential Discriminating Validity of CCNB1/PKD1 for IDD

**Figure 7:**
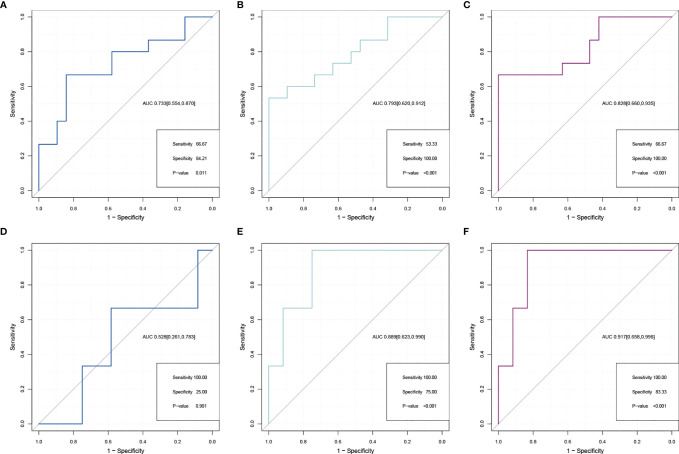
Comparison of ROC analysis between gene pair and separate gene in predicting IDD in the expanded dataset **(A–C)** and external validation dataset **(D–F)**. ROCs for *CCNB1* **(A, D)**, *PKD1* **(B, E)**, and *CCNB1*–*PKD1* pairs **(C, F)**.

After exploring the key gene pair (*CCNB1*–*PKD1*) and genes (*PKD1* and *CCNB1*), their distinguishing abilities were compared by using AUCs to evaluate the performance in differentiating the IDD and control cohorts based on ROC analysis. The AUCs of ROC curve analyses of the gene pair and genes were used to evaluate *CCNB1*–*PKD1* pair (AUC [95%CI] = 0.828 [0.660,0.935]), *PKD1* (AUC [95%CI] = 0.793 [0.620,0.912]), and *CCNB1* (AUC [95%CI] = 0.733 [0.554,0.870]) (**Figures 7A–C**). Compared to *CCNB1* and *PKD1*, the *CCNB1*–*PKD1* pair demonstrated the best distinguishing ability with the highest AUC value for effectively differentiating IDD from control samples. We next validated the gene pair (*CCNB1*–*PKD1*), *PKD1*, and *CCNB1* in the validation set. The AUCs [95%CI] for *CCNB1*–*PKD1*, *PKD1* and *CCNB1*, were 0.917 [0.658,0.996], 0.889 [0.623,0.990], and 0.528 [0.261,0.783]. Based on these results for the external validation dataset, this gene pair has excellent discriminating ability (**Figures 7D–E**). In the training and validation cohorts, the sensitivities and specificities of the *CCNB1*–*PKD1* pair were better than or equal to those of *PKD1* and *CCNB1* alone. The results of ROC curve analysis, including the AUCs, sensitivities, and specificities, are shown in **Supplementary Table S10**.

## Discussion

Although many treatments that are relatively effective for alleviating IDD progression have been developed, IDD diagnosis remains largely dependent on imaging and clinical symptoms, which render early IDD diagnosis and timely intervention difficult. Thus, identifying a potentially precise diagnostic signature and therapeutic targets may lead to new approaches for patients with IDD. A large volume of data indicates that oxidative stress promotes IDD development and that immune cells play vital roles in IDD progression. In this study, we evaluated a potential oxidative stress-related signature for IDD and predicted the mechanisms regulating its function and relationships with infiltrating immune cells.

We identified 18 significant De-OSRGs by analyzing GEO databases and assembled them into 13 gene pairs. Kyoto Encyclopedia of Genes and Genomes enrichment suggested that the 18 De-OSRGs were mainly enriched in MAPK signaling, phosphoinositide 3-kinase–protein kinase B signaling, FoxO signaling, and cellular senescence. Most of these pathways mediate intricate signal-transduction cascades associated with inflammation triggered by OS (34–37). In terms of cellular senescence, oxidative stress-induced injury continually disrupts the integrity of genes and proteins, leading to the gradual aging of organisms (38). GO annotation demonstrated enrichment of apoptosis-related BPs and multiple kinase-related MFs. As previously reported, IDD is a chronic, perplexing process that affects apoptosis and various kinase-mediated pathways (39–41).

In contrast to traditional concepts, pairwise gene comparisons and integration strategies offer substantial advantages. Calculating the OSRG pair signature was specifically based on the relative ranking of OSRG expression levels. These calculations enabled pairwise weight comparison that did not require data normalization and overcame the batch effects associated with assorted platforms (**Figures 3A, B**). Moreover, pairwise gene comparisons have become accessible based on studies that included multiple datasets (14), offering a tremendous advantage over current orthopedic practices. Our results showed that discrimination based on the *CCNB1*–*PKD1* gene pair was significantly better than that using individual genes, suggesting that the integration strategy was reliable and robust.

CCNB1, a member of the cyclin family of proteins, integrates with a cell cycle-dependent Ser/Thr kinase to stimulate the production of the maturation-promoting factor, which plays an indispensable role in mitosis (42). Previous *in vivo* data demonstrated that *CCNB1* is responsible for cellular proliferation and differentiation and that its abolition inhibited proliferation and promoted apoptosis in gonocytes and spermatogonia (43). Meng et al. (44) revealed that CCNB1 is an important regulator of G2/M phase of NP cells. Once G2/M phase was arrested, NP cell proliferation was blocked. Several lines of evidence have indicated that CCNB1 is useful as a diagnostic or prognostic biomarker in rhabdomyosarcoma, hepatocellular carcinoma, and meningioma (42, 45, 46). Based on these findings, CCNB1 protein may participate in IDD development by regulating NP cell proliferation.

*PKD1* encodes a serine/threonine kinase that belongs to the PKD family within the Ca^2+^/calmodulin-dependent protein kinase superfamily (47). PKD1, when activated by hormones, oxidative stress, and/or growth factors, participates in cell proliferation, migration, motility, apoptosis, membrane trafficking, epidermal-to-mesenchymal transition, and angiogenesis (48, 49). Data from previous knockout studies indicated that *PKD1* orchestrates a battery of intracellular signaling pathways, such as cyclic AMP production, autophagy inhibition, G-protein coupled receptor signaling, MAPK signaling, nuclear factor-kappa B signaling, and cell–extracellular matrix interactions (50, 51). Although these signaling pathways have been reported to be distinctly linked to various diseases, the exact role of PKD1 remains unclear, particularly in neoplasia. For example, it was reported that PKD1 was highly expressed in skin and pancreatic cancers where it acted as a tumor-promoting factor (52, 53). Normally, PKD1 promotes mouse fibroblast and human endothelial cell proliferation (54, 55). In contrast, PKD1 functions as a tumor suppressor in invasive breast and gastric cancers (56, 57). This dual role of PKD1 depends on the degree of inflammation, microenvironmental factors, and tissue type or phenotype (58). Considering that PKD1 typically participates in proliferation, PKD1 may regulate NP cell proliferation during IDD. Previous evidence, as well as our current results, indicate that under oxidative stress conditions, the *CCNB1*–*PKD1* pair participates in IDD by regulating NP cell proliferation or apoptosis *via* a signaling pathway(s), such as the MAPK, nuclear factor-kappa B, and/or calcium-ion pathways. Although both *CCNB1* and *PKD1* may promote IDD, further verification *via* experimentation and clinical studies is needed to support this hypothesis.

Considering the predictive role of the *CCNB1*–*PKD1* signature in IDD, we further clarified the regulatory ceRNA mechanism associated with this pair. Comprehensive analysis through online database predictions and expression validation with GEO datasets (conducted in this study) revealed two regulatory ceRNA axes as potentially associated with *CCNB1* and *PKD1* modulation by oxidative stress in NP cells: the *CCNB1*–*miR-212-3p*–*AC079834* and *PKD1*–miR-20b-5p–*AP000797* axes. Specifically, *AC079834* acts as a ceRNA sponge for *miR-212-3p*, which inhibits its binding to *CCNB1* mRNA. Similarly, *PKD1* mRNA binding to miR-20b-5p is prone to interference by *AP000797*, which sponges miR-20b-5p. There was no *CCNB1* related reports linked to ceRNA in IDD in the literature. In terms of *PKD1*, only Qu et al. (59) identified an IDD-associated miRNA, *hsa-miR-140*, which was regulated by three lncRNAs: *KCNQ1* opposite strand/antisense transcript 1, OIP5 antisense RNA 1 and UGDH antisense RNA 1. Of the 3 lncRNAs, the latter two could target several overlapping co-expressed genes, including forkhead box F1 and polycystin 1, transient receptor potential channel interacting (*PKD1*). However, these findings were not validated experimentally. Increasing evidence suggests that lncRNAs and miRNAs regulate pathophysiologic BPs linked to IDD, such as oxidative stress and apoptosis (20).

*MiR-212-3p* acts as an important regulator of various cancers by modulating cell proliferation, angiogenesis, and tumor invasion (60). *MiR-212-3p* expression is decreased in patients with bladder cancer. Our results indicate that *miR-212-3p* upregulation promoted *CCNB1* downregulation, whereas downregulating *AC079834* upregulated *miR-212-3p*, thereby accelerating IDD development by reversing the former process. Previously, *miR-20b-5p* was reported as upregulated and could be used as a biomarker for diagnosing breast cancer (61). Our results also showed that *miR-20b-5p* downregulation induced *PKD1* upregulation, which was attenuated by *AP000797*. However, the functional roles of the two regulatory axes regarding oxidative stress in IDD must be validated in experimental studies.

In addition to the abovementioned regulatory mechanisms, the pattern of immune infiltration is another critical concern. Our immune-infiltration analysis of the three De-OSRG pairs (consisting of five OSRGs) showed that resting memory CD4^+^ T cell and resting dendritic cell infiltration was decreased in IDD, whereas CD8^+^ T cell infiltration was increased. Notably, only the *CCNB1*–*PKD1* pair was synchronously associated with CD8^+^ T cells. During the degenerative process, substantial amounts of inflammatory cytokines and chemokines secreted by NP cells drive the activation and recruitment of immunocytes (including T cells and B cells) to further enhance the inflammatory cascade (62). For example, Shamji et al. reported that interleukin-17 released by Th17 lymphocytes was substantially expressed in degenerated and herniated human intervertebral disc tissues, which induced macrophage recruitment (63). North et al. revealed that T cells, including CD4^+^ and CD8^+^ T cells, were closely associated with NP extrusion (64). Another study showed that painful human intervertebral discs were highly infiltrated by regulatory T cells (65). Recent data showed that macrophages and CD8^+^ T cells were more prone to apoptosis in rat IDD models than in normal rats (27). These findings partially clarify the function of CD8^+^ T cells in IDD. Consistent with the results of these studies, our results suggest that an increase in CD8^+^ T cells plays a role in immune infiltration, NP cell damage, and apoptosis during IDD pathogenesis. We conducted experiments to predict the role of CD8^+^ T cells in human IDD. However, the correlation between the *CCNB1*–*PKD1* pair and CD8^+^ T cells should be further investigated. In addition, the functional relevance of this pair in regulation of CD8^+^ T cells and associated signaling in other diseases were also explored. For example, autosomal dominant polycystic kidney disease is presumably the direct consequence of mutations in *PKD1* or *PKD2*. Kleczko et al. (66) confirmed an increase in renal T cells associated with autosomal dominant polycystic kidney disease severity, specific activation of CD8^+^ T cells, and a functional role for these cells in inhibiting cystogenesis. Craven et al. (67) reported that *PKD1* was more frequently mutated in a group enriched in both CD8^+^ T cells and CD4 memory-activated T cells in triple-negative breast cancer. Li et al. (68) reported that four cyclin family genes, including *CCNB1*, were notably elevated in the early TNM stages in colon cancer and significantly correlated with overall survival. Moreover, they also found that the expression of *CCNB1* was positively correlated with tumor-killing immune cells, such as CD8^+^ T cells, which may prolong the survival time of patients with colon cancer. Zou et al. (69) reported that upregulated *CCNB1* was associated with poorer prognosis in patients with hepatocellular carcinoma. Notably, through immune infiltration analysis, they found that the expression level of *CCNB1* was positively correlated with the infiltrating levels of CD8^+^ T cells. Kao et al. (70) identified *CCNB1* as a shared human epithelial tumor-associated antigen recognized by T cells, such as CD8^+^ T cells. Latner et al. (71) elucidated that the expression of cell cycle regulatory genes, such as *CCNB1*, was enhanced in virus-specific memory CD8^+^ T cells. Moreover, *CCNB1* elicits T cell-dependent antibody responses not only in patients with cancer and premalignant disease, but also in healthy individuals (72, 73).

The current study had some limitations. For example, although we merged multiple datasets to increase the sample size, the reliability of our findings must be confirmed using additional datasets and clinical samples, although more samples may not be readily obtainable. Moreover, as our study involved retrospective analysis, the potential diagnostic values and exact functions of *CCNB1* and *PKD1* should be verified in *in vivo* and *in vitro* experiments. Additionally, the lack of *in situ* localization/validation of the data limits the generality of the current findings. Further experimental verification is needed to determine whether CD8^+^ T cell infiltration causes IDD or whether infiltration occurs after oxidative stress.

In conclusion, our results suggest that the *CCNB1*–*PKD1* gene pair is a robust, oxidative stress-related signature for identifying IDD. This gene pair may modulate oxidative stress, which leads to IDD, by regulating the *CCNB1*–*miR-212-3p*–*AC079834* and *PKD1*–*miR-20b-5p*–*AP000797* axes. The *CCNB1*–PKD1 signature was closely associated with CD8^+^ T-cell infiltration. This *CCNB1*–*PKD1* signature provides a novel perspective on the immune microenvironment in IDD and is a promising immune target in patients with IDD. In our further studies, we will analyze the relationship between immunity, oxidative stress, and metabolism in IDD.

## Data Availability Statement

The original contributions presented in the study are included in the article/**Supplementary Material**. Further inquiries can be directed to the corresponding author.

## Ethics Statement

The studies involving human participants were reviewed and approved by The Second Affiliated Hospital of Xi’an Jiaotong University. Written informed consent for participation was not required for this study in accordance with the national legislation and the institutional requirements.

## Author Contributions

SC and HPL collected the data and drafted the manuscript. SC and KY analyzed the data. HPL designed the study. All authors contributed to the article and approved the submitted version.

## Funding

This work was supported by the Social Development Science and Technology Project in Shaanxi Province (grant number 2021SF-172).

## Conflict of Interest

The authors declare that the research was conducted in the absence of any commercial or financial relationships that could be construed as a potential conflict of interest.

## Publisher’s Note

All claims expressed in this article are solely those of the authors and do not necessarily represent those of their affiliated organizations, or those of the publisher, the editors and the reviewers. Any product that may be evaluated in this article, or claim that may be made by its manufacturer, is not guaranteed or endorsed by the publisher.

## Abbreviations

BP, biological process; CCNB1, cyclin B1; ceRNA, competitive endogenous RNA; CI, confidence interval; DElncRNA, differentially expressed long non-coding RNA; DEmiRNA, differentially expressed microRNA; DEmRNA, differentially expressed messenger RNA; De-OSRG, differentially expressed oxidative stress-related gene; De-OSRGP, differentially expressed oxidative stress-related gene pair; GEO, Gene Expression Omnibus; GO, Gene Ontology; IDD, intervertebral disc degeneration; KEGG, Kyoto Encyclopedia of Genes and Genomes; LASSO, least absolute shrinkage and selection operator; lncRNA, long non-coding RNA; MAPK, mitogen-activated protein kinase; MF, molecular function; miRNA, microRNA; mRNA, messenger RNA; NP, nucleus pulposus; OS, oxidative stress; OSRG, oxidative stress-related gene; OSRGP, oxidative stress-related gene pair; PKD1, protein kinase D1; ROC, receiver operating characteristic; ROS, reactive oxygen species; SVM, support vector machine.
