# Supplementary material for: An Oxidative Stress-Related Gene Pair (CCNB1/PKD1), Competitive Endogenous RNAs, and Immune-Infiltration Patterns Potentially Regulate Intervertebral Disc Degeneration Development
Source: Front Immunol. 2021 Nov 9;12:765382. doi: 10.3389/fimmu.2021.765382 (PMC8630707; doi:10.3389/fimmu.2021.765382)
Supplement: Supplementary file 1 [file DataSheet_1.docx]

Supplementary Material

# Supplementary Table S1–S11

**Supplementary Table S1.** Details of the GEO datasets analyzed in this study.

| **GEO Datasets** | **Set for** | **Groups** | **N** | **Platform infor.** | **URL** |
| --- | --- | --- | --- | --- | --- |
| GSE116726 | miRNA |  |  | GPL20712 | https://www.ncbi.nlm.nih.gov/geo/query/acc.cgi?acc=GSE116726 |
|  |  | Control | 3 |  |  |
|  |  | IDD | 3 |  |  |
| GSE56081 | lncRNA & mRNA |  |  | GPL15314 | https://www.ncbi.nlm.nih.gov/geo/query/acc.cgi?acc=GSE56081 |
|  |  | Control | 5 |  |  |
|  |  | IDD | 5 |  |  |
| GSE70362 | mRNA |  |  | GPL17810 | https://www.ncbi.nlm.nih.gov/geo/query/acc.cgi?acc=GSE70362 |
|  |  | Control | 14 |  |  |
|  |  | IDD | 10 |  |  |
| GSE15227 | mRNA |  |  | GPL1352 | https://www.ncbi.nlm.nih.gov/geo/query/acc.cgi?acc=GSE15227 |
|  |  | Control | 12 |  |  |
|  |  | IDD | 3 |  |  |

**Abbreviations:** GEO: Gene Expression Omnibus (<https://www.ncbi.nlm.nih.gov/geo>). Platform infor., Platform information. IDD, Intervertebral disc degeneration.

**Supplementary Table S2.** URL for the databases utilized in this study.

| **Database** | **URL** |
| --- | --- |
| GEO | https://www.ncbi.nlm.nih.gov/geo/ |
| GeneCards | https://www.genecards.org/ |
| MiRDB | http://mirdb.org/ |
| miRTarBase | http://mirtarbase.mbc.nctu.edu.tw/php/index.php |
| TargetScan | http://www.targetscan.org/vert_72/ |
| miRcode | http://www.mircode.org/ |

**Supplementary Table S3.** Clinical information contained in each data set.

| **Data sets** | **Cohorts** | **Samples** | **Gender(F/M)** | **Age (average, years)** | **Address** |
| --- | --- | --- | --- | --- | --- |
| GSE116726 | Control | 3 | 0/3 | 56 | China |
|  | IDD | 3 | 0/3 | 56 |  |
| GSE56081 | Control | 5 | 1/4 | 40.8 | China |
|  | IDD | 5 | 2/3 | 36.8 |  |
| GSE70362 | Control | 14 | 7/7 | 48.9 | Ireland |
|  | IDD | 10 | 3/7 | 74.8 |  |
| GSE15227 | Control | 12 | NA/NA | NA | USA |
|  | IDD | 3 | NA/NA | NA |  |

**Abbreviation**: F, Female; M, Male; NA, not applicable.

**Supplementary Table S4.** Symbols of all 1399 OS-related genes considered in this study.

| A2M | AARS2 | ABCA1 | ABCB1 | ABCC1 | ABCC2 | ABCC3 | ABCC8 | ABCD1 | ABCG2 |
| --- | --- | --- | --- | --- | --- | --- | --- | --- | --- |
| ABL1 | ACACA | ACAD8 | ACAD9 | ACADL | ACADM | ACADS | ACADVL | ACE | ACE2 |
| ACHE | ACO1 | ACO2 | ACOX1 | ACOX2 | ACP1 | ACSL4 | ACTA1 | ACTB | ACTG1 |
| ACTN2 | ACTN4 | ADA | ADAM10 | ADAM17 | ADAMTS13 | ADCY10 | ADCYAP1 | ADH1A | ADH1C |
| ADH5 | ADIPOQ | ADM | ADORA2A | ADPRS | ADRB1 | ADRB2 | ADRB3 | ADSL | AGER |
| AGRN | AGT | AGTR1 | AHR | AHSP | AIF1 | AIFM1 | AKAP9 | AKR1A1 | AKR1B1 |
| AKT1 | AKT2 | ALAD | ALB | ALDH1A1 | ALDH2 | ALDH3A1 | ALDH3A2 | ALDH3B1 | ALDH9A1 |
| ALOX12 | ALOX15 | ALOX5 | ALS3 | ALS7 | AMPD1 | ANG | ANGPT1 | ANGPT2 | ANK2 |
| ANXA11 | ANXA2 | ANXA5 | AOC3 | AOX1 | APAF1 | APC | APEX1 | APOA1 | APOB |
| APOE | APOH | APP | AQP1 | AQP4 | AR | AREG | ARG1 | ARG2 | ARNT |
| ASL | ASPA | ASS1 | ATF2 | ATF3 | ATF4 | ATF6 | ATG5 | ATM | ATP13A2 |
| ATP2A2 | ATP5F1A | ATP5PD | ATR | ATXN1 | ATXN2 | ATXN3 | ATXN8OS | AURKA | AVP |
| B2M | BACE1 | BACH1 | BACH2 | BAD | BAG3 | BAK1 | BAX | BBC3 | BCHE |
| BCL2 | BCL2A1 | BCL2L1 | BCL2L11 | BCL6 | BCR | BDKRB2 | BDNF | BECN1 | BGLAP |
| BIRC5 | BLK | BLOC1S1 | BLVRB | BMP2 | BMP4 | BMP6 | BRAF | BRCA1 | BRCA2 |
| BRF2 | BSG | BTD | C12orf65 | C1QBP | C3 | C4A | C4B | C5 | C5AR1 |
| C9orf72 | CACNA1A | CACNA1C | CACNA1S | CACNA2D1 | CACNB4 | CALB1 | CALB2 | CALCA | CALM1 |
| CALM2 | CALM3 | CALR | CAMK2G | CAMK4 | CAMKK2 | CAMP | CANX | CAPN2 | CAPN3 |
| CARS2 | CASP1 | CASP2 | CASP3 | CASP4 | CASP7 | CASP8 | CASP9 | CASQ2 | CAT |
| CAV1 | CAV3 | CBS | CCK | CCL11 | CCL2 | CCL3 | CCL4 | CCL5 | CCN2 |
| CCNA2 | CCNB1 | CCND1 | CCNF | CCR5 | CCR6 | CCR7 | CCS | CD274 | CD28 |
| CD34 | CD36 | CD38 | CD4 | CD40 | CD40LG | CD44 | CD46 | CD55 | CD69 |
| CD79A | CD80 | CD86 | CDC25C | CDC42 | CDH1 | CDH2 | CDH5 | CDK1 | CDK2 |
| CDK4 | CDK5 | CDK6 | CDKN1A | CDKN1B | CDKN2A | CDKN2B | CDKN3 | CEBPB | CFAP410 |
| CFH | CFI | CFLAR | CFTR | CHAT | CHCHD10 | CHCHD2 | CHEK1 | CHGA | CHKA |
| CHKB | CHMP2B | CHUK | CIITA | CLEC4A | CLIC1 | CLU | CNR1 | CNTF | COA8 |
| COL2A1 | COMT | COQ2 | COX15 | COX5A | COX6B1 | CP | CPOX | CPQ | CPT1A |
| CPT1B | CPT2 | CR1 | CR2 | CRAT | CREB1 | CREBBP | CRH | CRHR1 | CRP |
| CRYAA | CRYAB | CS | CSF1 | CSF2 | CSF3 | CSK | CST3 | CTLA4 | CTNNB1 |
| CTSB | CTSD | CTSG | CTTN | CUL1 | CUL3 | CX3CR1 | CXCL1 | CXCL10 | CXCL12 |
| CXCL16 | CXCL2 | CXCL8 | CXCL9 | CXCR1 | CXCR3 | CXCR4 | CYB5A | CYB5R3 | CYBA |
| CYBB | CYC1 | CYCS | CYGB | CYP11A1 | CYP11B2 | CYP17A1 | CYP19A1 | CYP1A1 | CYP1A2 |
| CYP1B1 | CYP20A1 | CYP21A2 | CYP27A1 | CYP2A6 | CYP2B6 | CYP2C19 | CYP2C8 | CYP2C9 | CYP2D6 |
| CYP2E1 | CYP3A4 | CYP3A5 | CYP4F2 | DAO | DAPK1 | DAXX | DBH | DCTN1 | DDAH1 |
| DDAH2 | DDC | DDIT3 | DECR1 | DEPDC5 | DES | DGKQ | DHCR24 | DHFR | DIABLO |
| DLD | DLG4 | DLST | DMD | DMPK | DNAH8 | DNAJB1 | DNASE1 | DNM1L | DNM2 |
| DNMT1 | DRD1 | DRD2 | DRD3 | DRD4 | DRD5 | DSP | DSPP | DUOX1 | DUSP1 |
| DUSP19 | DYNC1H1 | DYNLL1 | DYRK1A | DYSF | E2F1 | ECE1 | ECHS1 | EDN1 | EDNRA |
| EDNRB | EEF1A1 | EEF2 | EGF | EGFR | EGR1 | EHHADH | EIF2AK1 | EIF2AK2 | EIF2AK3 |
| EIF2AK4 | EIF2B3 | EIF2B4 | EIF2S1 | EIF4E | EIF4EBP1 | EIF4G1 | ELAC2 | ELANE | ELAVL1 |
| ELK1 | ELN | ENC1 | ENDOG | ENG | ENO1 | ENO2 | EP300 | EPAS1 | EPHA3 |
| EPHA4 | EPHX1 | EPHX2 | EPO | EPRS1 | EPX | ERBB2 | ERBB4 | ERCC6 | ERCC8 |
| ERN1 | ERO1A | ESR1 | ESR2 | ETFA | ETFB | ETFDH | ETS1 | EZH2 | F2 |
| F3 | F5 | F8 | FAAH | FABP1 | FADD | FAM120A | FANCD2 | FARS2 | FAS |
| FASLG | FASN | FCGR2A | FCGR2B | FCGR3A | FCGR3B | FDXR | FECH | FGF1 | FGF2 |
| FGF7 | FGFR1 | FH | FIG4 | FIS1 | FKBP1B | FKBP5 | FKRP | FLT1 | FMO1 |
| FMO2 | FMO3 | FMO4 | FMR1 | FN1 | FOS | FOXJ1 | FOXM1 | FOXO1 | FOXO3 |
| FOXO4 | FOXP3 | FRZB | FTH1 | FTL | FUS | FXN | FYN | G3BP1 | G6PD |
| GAA | GAD1 | GADD45A | GADD45B | GADD45G | GAL | GAP43 | GAPDH | GBA | GCDH |
| GCH1 | GCLC | GCLM | GDF15 | GDNF | GFAP | GFER | GFM1 | GFM2 | GGT1 |
| GH1 | GHRL | GIGYF2 | GJA1 | GLA | GLE1 | GLO1 | GLRX | GLRX2 | GLS |
| GLS2 | GLT8D1 | GLUD1 | GLUD2 | GLUL | GNAS | GP1BA | GPT | GPX1 | GPX2 |
| GPX3 | GPX4 | GPX5 | GPX7 | GPX8 | GRB2 | GRIA1 | GRIN1 | GRIN2A | GRIN2B |
| GRM1 | GRM5 | GRN | GSK3B | GSN | GSR | GSS | GSTA1 | GSTA2 | GSTA4 |
| GSTM1 | GSTM2 | GSTM3 | GSTM4 | GSTM5 | GSTO1 | GSTO2 | GSTP1 | GSTT1 | GTPBP3 |
| GYG1 | GZMB | H19 | H2AC18 | H2AX | H2BC21 | H3C14 | H4-16 | H6PD | HADH |
| HADHA | HADHB | HAMP | HAO1 | HBA1 | HBB | HBEGF | HBG2 | HCRT | HDAC1 |
| HDAC2 | HDAC9 | HERPUD1 | HFE | HGF | HIF1A | HK1 | HK2 | HLA-A | HLA-B |
| HLA-DRA | HLA-DRB1 | HMGB1 | HMGCL | HMGCR | HMOX1 | HMOX2 | HNF1A | HNF4A | HNRNPA1 |
| HP | HPRT1 | HPSE | HPX | HRAS | HRH1 | HRH2 | HSD17B10 | HSD17B4 | HSF1 |
| HSP90AA1 | HSP90AB1 | HSP90B1 | HSPA14 | HSPA1A | HSPA1B | HSPA4 | HSPA5 | HSPA6 | HSPA8 |
| HSPA9 | HSPB1 | HSPB2 | HSPD1 | HSPG2 | HTR1A | HTR2A | HTR2C | HTR3A | HTRA2 |
| HTT | HYOU1 | IAPP | ICAM1 | IDH1 | IDH2 | IDO1 | IFNA1 | IFNAR1 | IFNB1 |
| IFNG | IGF1 | IGF1R | IGF2 | IGF2BP1 | IGF2BP2 | IGF2R | IKBKB | IKBKG | IL10 |
| IL11 | IL12B | IL13 | IL15 | IL16 | IL17A | IL18 | IL1A | IL1B | IL1R1 |
| IL1RAPL2 | IL1RN | IL2 | IL23A | IL2RA | IL2RB | IL3 | IL33 | IL4 | IL5 |
| IL6 | IL6R | INS | INSR | IRAK1 | IREB2 | IRF1 | IRF5 | IRS1 | ISCU |
| ISG15 | ITGA2 | ITGAL | ITGAM | ITGB1 | ITGB2 | ITGB3 | ITIH4 | ITPR1 | JAK1 |
| JAK2 | JAZF1 | JUN | JUNB | KCNE1 | KCNE2 | KCNH2 | KCNJ2 | KCNJ5 | KCNMA1 |
| KCNQ1 | KCNT1 | KDR | KEAP1 | KIAA0319L | KIF1B | KIT | KL | KLF2 | KLF4 |
| KLRK1 | KNG1 | KRAS | KRIT1 | KRT18 | KRT8 | LAMP1 | LAMP2 | LANCL1 | LBR |
| LCAT | LCK | LCN2 | LDLR | LEP | LEPQTL1 | LGALS1 | LGALS3 | LIN28B | LMNA |
| LOC110806262 | LOC110973015 | LOC111365141 | LONP1 | LOX | LPA | LPL | LPO | LRPPRC | LRRK2 |
| LTA | LTF | LYN | LYRM4 | MALAT1 | MAOA | MAOB | MAP2 | MAP2K1 | MAP2K3 |
| MAP2K4 | MAP2K6 | MAP2K7 | MAP3K1 | MAP3K11 | MAP3K5 | MAP3K7 | MAPK1 | MAPK10 | MAPK11 |
| MAPK12 | MAPK13 | MAPK14 | MAPK3 | MAPK7 | MAPK8 | MAPK8IP1 | MAPK9 | MAPKAPK2 | MAPKAPK3 |
| MAPKAPK5 | MAPT | MATR3 | MB | MBL2 | MBP | MCL1 | MCU | MDH1 | MDH2 |
| MDM2 | MECOM | MECP2 | MET | MFN2 | MGMT | MGST1 | MICB | MIF | MIR106B |
| MIR107 | MIR122 | MIR125A | MIR126 | MIR132 | MIR133B | MIR142 | MIR143 | MIR144 | MIR145 |
| MIR146A | MIR148B | MIR152 | MIR155 | MIR17 | MIR181A1 | MIR181A2 | MIR181C | MIR184 | MIR185 |
| MIR195 | MIR199A1 | MIR19A | MIR200B | MIR200C | MIR203A | MIR20A | MIR21 | MIR210 | MIR214 |
| MIR22 | MIR221 | MIR222 | MIR223 | MIR23A | MIR23B | MIR24-1 | MIR24-2 | MIR25 | MIR27A |
| MIR29A | MIR34A | MIR34C | MIR433 | MIR9-1 | MIR92A1 | MIR93 | MKI67 | MLYCD | MMD |
| MME | MMP1 | MMP13 | MMP14 | MMP2 | MMP3 | MMP7 | MMP8 | MMP9 | MPO |
| MRAP | MRPL44 | MRPS14 | MRPS16 | MRPS22 | MRPS34 | MSH2 | MSN | MSR1 | MSRA |
| MSRB1 | MSRB2 | MSRB3 | MT3 | MTA1 | MT-ATP6 | MT-CO1 | MT-CO2 | MT-CO3 | MT-CYB |
| MTFMT | MTHFR | MT-ND1 | MT-ND2 | MT-ND3 | MT-ND4 | MT-ND5 | MT-ND6 | MTO1 | MTOR |
| MTR | MT-TK | MT-TL1 | MTTP | MUC1 | MUC5AC | MUTYH | MYC | MYD88 | MYH6 |
| MYH7 | MYLK | MYO9A | NAGS | NAMPT | NAT2 | NCAM1 | NCF1 | NCF2 | NCF4 |
| NDRG1 | NDUFA1 | NDUFA10 | NDUFA12 | NDUFA13 | NDUFA6 | NDUFA9 | NDUFAF2 | NDUFB8 | NDUFB9 |
| NDUFS1 | NDUFS2 | NDUFS3 | NDUFS4 | NDUFS6 | NDUFS7 | NDUFS8 | NDUFV1 | NDUFV2 | NEAT1 |
| NEDD8 | NEFH | NEFL | NEIL1 | NEK1 | NES | NFE2L1 | NFE2L2 | NFKB1 | NFKBIA |
| NFU1 | NGB | NGF | NGFR | NLRP1 | NLRP3 | NME1 | NOD2 | NOL3 | NOS1 |
| NOS1AP | NOS2 | NOS3 | NOSIP | NOSTRIN | NOTCH1 | NOX1 | NOX4 | NPM1 | NPPA |
| NPPB | NPY | NQO1 | NQO2 | NR1H2 | NR1H4 | NR2C2 | NR3C1 | NR3C2 | NR4A2 |
| NRAS | NRF1 | NRG1 | NTF3 | NTF4 | NTHL1 | NTRK1 | NTRK2 | NTS | NUDT1 |
| ODC1 | OGDH | OGG1 | OLR1 | OPA1 | OPRD1 | OPRM1 | OPTN | OSER1 | OSGIN1 |
| OSGIN2 | OSM | OTC | OXA1L | OXR1 | OXSR1 | OXT | OXTR | P4HB | PAH |
| PARK10 | PARK12 | PARK16 | PARK21 | PARK7 | PARP1 | PC | PCNA | PDCD1 | PDE4A |
| PDE5A | PDGFB | PDGFRB | PDGFRL | PDHA1 | PDIA2 | PDIA3 | PDK1 | PDLIM4 | PDYN |
| PECAM1 | PENK | PEPD | PEX11B | PEX12 | PEX5 | PF4 | PFKM | PFN1 | PGAM5 |
| PGD | PGK1 | PHYH | PIGA | PIK3C2A | PIK3C3 | PIK3CA | PIK3CB | PIK3CG | PIK3R1 |
| PIK3R2 | PINK1 | PKD1 | PKLR | PKM | PKP2 | PLA2G2A | PLA2G4A | PLA2G6 | PLA2G7 |
| PLAT | PLAU | PLAUR | PLCB1 | PLCG1 | PLD1 | PLG | PML | PNKP | PNPT1 |
| POLG | POMC | PON1 | PON2 | PON3 | POR | POU5F1 | PPARA | PPARD | PPARG |
| PPARGC1A | PPIA | PPIF | PPIG | PPOX | PPP1R15A | PPP3CA | PPP5C | PRDM10 | PRDX1 |
| PRDX2 | PRDX3 | PRDX4 | PRDX5 | PRDX6 | PRKAA1 | PRKAA2 | PRKAB1 | PRKCA | PRKCB |
| PRKCD | PRKCG | PRKCZ | PRKD1 | PRKD2 | PRKG1 | PRKN | PRL | PRNP | PRODH |
| PRPH | PSEN1 | PSEN2 | PSIP1 | PTEN | PTGIS | PTGS1 | PTGS2 | PTK2 | PTK2B |
| PTPA | PTPN1 | PTPN11 | PTPN22 | PTPN3 | PTPRC | PTS | PTX3 | PVALB | PXN |
| PYCR1 | PYCR2 | PYGM | QDPR | RAB5A | RAC1 | RAC2 | RAD51 | RAF1 | RAG2 |
| RARA | RB1 | RBP4 | RCAN1 | RELA | REN | REST | RETN | RHOA | RHOD |
| RNASE3 | RNF112 | ROCK1 | RORA | RPA1 | RPS27A | RPS6KA5 | RPS6KB1 | RPTOR | RRM2B |
| RTN4 | RUNX2 | RXRA | RYR1 | RYR2 | RYR3 | S100A8 | S100A9 | S100B | SCARA3 |
| SCARB1 | SCGB1A1 | SCN2A | SCN4A | SCN4B | SCN5A | SCO1 | SCO2 | SCP2 | SDC1 |
| SDHA | SDHAF1 | SDHAF2 | SDHB | SDHC | SDHD | SELE | SELENOK | SELENON | SELENOP |
| SELENOT | SELL | SELP | SENP3 | SERPINA1 | SERPINA3 | SERPINE1 | SERPINF1 | SERPINH1 | SESN1 |
| SESN2 | SET | SETD2 | SETX | SFTPB | SFTPD | SFXN4 | SGCB | SGK1 | SHC1 |
| SIAH1 | SIGMAR1 | SIL1 | SIRT1 | SIRT2 | SIRT3 | SIRT6 | SLC11A2 | SLC17A5 | SLC18A2 |
| SLC18A3 | SLC19A3 | SLC1A1 | SLC1A2 | SLC1A3 | SLC22A5 | SLC25A1 | SLC25A13 | SLC25A20 | SLC25A27 |
| SLC25A3 | SLC25A4 | SLC2A1 | SLC2A4 | SLC40A1 | SLC4A1 | SLC5A7 | SLC6A2 | SLC6A3 | SLC6A4 |
| SLC7A1 | SLC7A11 | SLC8A1 | SLPI | SMAD2 | SMAD3 | SMAD4 | SMARCA4 | SMPD1 | SNAP25 |
| SNCA | SNCAIP | SNCB | SNTA1 | SOCS1 | SOCS3 | SOD1 | SOD2 | SOD3 | SORCS2 |
| SORD | SORL1 | SOX2 | SP1 | SPARC | SPP1 | SPR | SQSTM1 | SRC | SREBF1 |
| SRF | SRXN1 | SST | STAT1 | STAT3 | STAT4 | STIP1 | STK11 | STK24 | STK25 |
| STK39 | STK4 | STUB1 | SULT1A3 | SUMO1 | SUMO2 | SUOX | SYK | SYP | TAC1 |
| TACO1 | TACR1 | TAF15 | TALDO1 | TARDBP | TARS2 | TAT | TAZ | TBK1 | TBP |
| TCF7L2 | TECRL | TEK | TERT | TF | TFAM | TFEB | TFRC | TGFA | TGFB1 |
| TGFB2 | TGFB3 | TGFBR1 | TGFBR2 | TGM2 | TH | THBD | THBS1 | TIA1 | TIMP1 |
| TIMP2 | TJP1 | TLR2 | TLR3 | TLR4 | TLR5 | TLR6 | TLR7 | TLR8 | TLR9 |
| TMEM161A | TNF | TNFAIP3 | TNFRSF10A | TNFRSF10B | TNFRSF11B | TNFRSF1A | TNFRSF1B | TNFSF10 | TNFSF11 |
| TNFSF4 | TNIP1 | TOP1 | TOR1A | TP53 | TP53INP1 | TP73 | TPH1 | TPI1 | TPK1 |
| TPM1 | TPO | TPPP3 | TPT1 | TRAF2 | TRAP1 | TRDN | TREM2 | TREX1 | TRIM21 |
| TRMT10C | TRPA1 | TRPM2 | TRPV1 | TRPV4 | TSC1 | TSC2 | TSFM | TSPO | TTN |
| TTPA | TTR | TUBA1B | TUFM | TXN | TXN2 | TXNIP | TXNRD1 | TXNRD2 | TYK2 |
| TYMP | TYR | TYRP1 | UBC | UBE2D2 | UBE2L3 | UBQLN1 | UBQLN2 | UBQLN4 | UCHL1 |
| UCN | UCN2 | UCP1 | UCP2 | UCP3 | UGT1A1 | UNC13A | UNG | UTRN | VAPB |
| VARS2 | VASP | VCAM1 | VCL | VCP | VDAC1 | VDR | VEGFA | VEGFC | VHL |
| VIM | VIP | VIPR1 | VKORC1L1 | VNN1 | VPS13C | VTN | VWF | WRN | XBP1 |
| XDH | XIAP | XRCC1 | XRCC5 | XRCC6 | YAP1 | YBX1 | ZC3H12A | ZFAND1 |  |

**Supplementary table S5**. List of the R software packages used for analysis in this study.

| **Analysis** | **R Packages** |
| --- | --- |
| Normalization | “sva” |
| Difference analysis | “limma” |
| Heat map | “pheatmap” |
| Volcano map | “ggplot2” |
| GO, KEGG | “clusterProfiler”, “org.Hs.eg.db”, “enrichplot”, “ggplot2” |
| Lasso | “glmnet” |
| SVM | “caret” |
| Immune infiltration | “CIBERSORT” (source), “parallel”, “preprocessCore”, “e1071” |
| Violin diagram | “vioplot” |
| Correlation | “corrplot”, “ggplot2”, “tidyverse”, “ggsci” |
| ROC | “pROC” |

**Supplementary Table S6.** Detailed GO descriptions and related OS gene names.

| **GO** | **Description** | **P-value** | **Qvalue** | **Gene Names** | **Count** |
| --- | --- | --- | --- | --- | --- |
| BP | response to acid chemical | 0.0006 | 0.0003 | ACACA/ATF2/FOXO1/KLF4/NTRK2/CCNB1 | 6 |
| BP | response to mechanical stimulus | 0.0006 | 0.0003 | IRF1/KCNJ2/PKD1/TGFBR2/CCNB1 | 5 |
| BP | regulation of neuron apoptotic process | 0.0006 | 0.0003 | ATF2/LANCL1/MAP3K11/MCL1/NTRK2 | 5 |
| BP | neuron apoptotic process | 0.0008 | 0.0005 | ATF2/LANCL1/MAP3K11/MCL1/NTRK2 | 5 |
| BP | peptidyl-serine phosphorylation | 0.0017 | 0.0009 | DGKQ/NTRK2/PKD1/TGFBR2/CCNB1 | 5 |
| BP | cellular response to oxidative stress | 0.0017 | 0.0009 | FOXO1/KLF4/LANCL1/MCL1/TXNRD1 | 5 |
| BP | regulation of neuron death | 0.0018 | 0.001 | ATF2/LANCL1/MAP3K11/MCL1/NTRK2 | 5 |
| BP | peptidyl-serine modification | 0.0018 | 0.001 | DGKQ/NTRK2/PKD1/TGFBR2/CCNB1 | 5 |
| BP | neuron death | 0.0023 | 0.0013 | ATF2/LANCL1/MAP3K11/MCL1/NTRK2 | 5 |
| BP | positive regulation of neuron apoptotic process | 0.0029 | 0.0016 | ATF2/MAP3K11/MCL1 | 3 |
| BP | regulation of angiogenesis | 0.003 | 0.0016 | ATF2/ERBB2/KLF4/TGFBR2/VEGFC | 5 |
| BP | regulation of vasculature development | 0.0043 | 0.0023 | ATF2/ERBB2/KLF4/TGFBR2/VEGFC | 5 |
| BP | cellular response to acid chemical | 0.0043 | 0.0023 | ACACA/KLF4/NTRK2/CCNB1 | 4 |
| BP | response to oxidative stress | 0.0051 | 0.0028 | FOXO1/KLF4/LANCL1/MCL1/TXNRD1 | 5 |
| BP | protein autophosphorylation | 0.0057 | 0.0031 | ERBB2/MAP3K11/NTRK2/VEGFC | 4 |
| BP | negative regulation of muscle adaptation | 0.0057 | 0.0031 | FOXO1/KLF4 | 2 |
| BP | cellular response to ketone | 0.0071 | 0.0039 | ACACA/FOXO1/KLF4 | 3 |
| BP | positive regulation of neuron death | 0.0071 | 0.0039 | ATF2/MAP3K11/MCL1 | 3 |
| BP | negative regulation of anoikis | 0.0084 | 0.0046 | MCL1/NTRK2 | 2 |
| BP | lymph vessel morphogenesis | 0.01 | 0.0055 | PKD1/VEGFC | 2 |
| BP | regulation of leukocyte cell-cell adhesion | 0.0113 | 0.0062 | ERBB2/IRF1/KLF4/TGFBR2 | 4 |
| BP | regulation of anoikis | 0.013 | 0.0071 | MCL1/NTRK2 | 2 |
| BP | negative regulation of leukocyte cell-cell adhesion | 0.013 | 0.0071 | ERBB2/IRF1/KLF4 | 3 |
| BP | positive regulation of protein serine/threonine kinase activity | 0.013 | 0.0071 | ERBB2/MAP3K11/PKD1/CCNB1 | 4 |
| BP | lymph vessel development | 0.013 | 0.0071 | PKD1/VEGFC | 2 |
| BP | amelogenesis | 0.013 | 0.0071 | ATF2/FOXO1 | 2 |
| BP | leukocyte cell-cell adhesion | 0.013 | 0.0071 | ERBB2/IRF1/KLF4/TGFBR2 | 4 |
| BP | digestive tract development | 0.013 | 0.0071 | PKD1/TGFBR2/CCNB1 | 3 |
| BP | regulation of oxidative stress-induced neuron death | 0.013 | 0.0071 | LANCL1/MCL1 | 2 |
| BP | regulation of T cell differentiation | 0.0132 | 0.0072 | ERBB2/IRF1/TGFBR2 | 3 |
| BP | neuron death in response to oxidative stress | 0.0132 | 0.0072 | LANCL1/MCL1 | 2 |
| BP | digestive system development | 0.0147 | 0.0081 | PKD1/TGFBR2/CCNB1 | 3 |
| BP | phosphatidylinositol 3-kinase signaling | 0.0148 | 0.0081 | ERBB2/KLF4/NTRK2 | 3 |
| BP | regulation of alpha-beta T cell proliferation | 0.0148 | 0.0081 | IRF1/TGFBR2 | 2 |
| BP | positive regulation of cyclin-dependent protein serine/threonine kinase activity | 0.0154 | 0.0084 | PKD1/CCNB1 | 2 |
| BP | regulation of T cell proliferation | 0.0159 | 0.0087 | ERBB2/IRF1/TGFBR2 | 3 |
| BP | alpha-beta T cell proliferation | 0.0165 | 0.0091 | IRF1/TGFBR2 | 2 |
| BP | response to oxygen levels | 0.0167 | 0.0091 | FOXO1/TGFBR2/VEGFC/CCNB1 | 4 |
| BP | anoikis | 0.0167 | 0.0091 | MCL1/NTRK2 | 2 |
| BP | positive regulation of cyclin-dependent protein kinase activity | 0.0169 | 0.0092 | PKD1/CCNB1 | 2 |
| BP | regulation of cell-cell adhesion | 0.0169 | 0.0092 | ERBB2/IRF1/KLF4/TGFBR2 | 4 |
| BP | regulation of lymphocyte differentiation | 0.0171 | 0.0094 | ERBB2/IRF1/TGFBR2 | 3 |
| BP | regulation of lipid metabolic process | 0.0171 | 0.0094 | ACACA/DGKQ/KLF4/TXNRD1 | 4 |
| BP | protein tetramerization | 0.0171 | 0.0094 | ACACA/KCNJ2/PKD1 | 3 |
| BP | response to fluid shear stress | 0.0171 | 0.0094 | KLF4/PKD1 | 2 |
| BP | negative regulation of protein phosphorylation | 0.0177 | 0.0097 | DGKQ/FOXO1/KLF4/CCNB1 | 4 |
| BP | negative regulation of cell-cell adhesion | 0.0177 | 0.0097 | ERBB2/IRF1/KLF4 | 3 |
| BP | phosphatidylinositol-mediated signaling | 0.0177 | 0.0097 | ERBB2/KLF4/NTRK2 | 3 |
| BP | cell fate determination | 0.0177 | 0.0097 | KLF4/MCL1 | 2 |
| BP | T cell proliferation | 0.0177 | 0.0097 | ERBB2/IRF1/TGFBR2 | 3 |
| BP | inositol lipid-mediated signaling | 0.0177 | 0.0097 | ERBB2/KLF4/NTRK2 | 3 |
| BP | regulation of G1/S transition of mitotic cell cycle | 0.0177 | 0.0097 | KLF4/PKD1/CCNB1 | 3 |
| BP | gastrulation | 0.0177 | 0.0097 | KLF4/TGFBR2/TXNRD1 | 3 |
| BP | electron transport chain | 0.0177 | 0.0097 | TXNRD1/CCNB1/COX15 | 3 |
| BP | response to ketone | 0.0193 | 0.0106 | ACACA/FOXO1/KLF4 | 3 |
| BP | regulation of reactive oxygen species metabolic process | 0.0194 | 0.0106 | FOXO1/KLF4/TGFBR2 | 3 |
| BP | regulation of small molecule metabolic process | 0.0194 | 0.0106 | ACACA/DGKQ/FOXO1/CCNB1 | 4 |
| BP | negative regulation of T cell differentiation | 0.0194 | 0.0106 | ERBB2/IRF1 | 2 |
| BP | regeneration | 0.0194 | 0.0106 | KLF4/TGFBR2/CCNB1 | 3 |
| BP | negative regulation of phosphorylation | 0.0199 | 0.0109 | DGKQ/FOXO1/KLF4/CCNB1 | 4 |
| BP | regulation of cell cycle G1/S phase transition | 0.0199 | 0.0109 | KLF4/PKD1/CCNB1 | 3 |
| BP | positive regulation of angiogenesis | 0.0201 | 0.011 | KLF4/TGFBR2/VEGFC | 3 |
| BP | regulation of gluconeogenesis | 0.0206 | 0.0113 | DGKQ/FOXO1 | 2 |
| BP | regulation of lymphocyte proliferation | 0.0206 | 0.0113 | ERBB2/IRF1/TGFBR2 | 3 |
| BP | regulation of mononuclear cell proliferation | 0.0206 | 0.0113 | ERBB2/IRF1/TGFBR2 | 3 |
| BP | regulation of leukocyte proliferation | 0.0241 | 0.0132 | ERBB2/IRF1/TGFBR2 | 3 |
| BP | fat cell differentiation | 0.0241 | 0.0132 | ATF2/FOXO1/KLF4 | 3 |
| BP | negative regulation of lymphocyte differentiation | 0.025 | 0.0137 | ERBB2/IRF1 | 2 |
| BP | positive regulation of vasculature development | 0.0254 | 0.0139 | KLF4/TGFBR2/VEGFC | 3 |
| BP | regulation of cardiac muscle cell proliferation | 0.0254 | 0.0139 | TGFBR2/CCNB1 | 2 |
| BP | cellular response to fatty acid | 0.0254 | 0.0139 | ACACA/CCNB1 | 2 |
| BP | response to alcohol | 0.0254 | 0.0139 | ACACA/KLF4/TGFBR2 | 3 |
| BP | cell cycle arrest | 0.0263 | 0.0144 | IRF1/PKD1/CCNB1 | 3 |
| BP | muscle cell proliferation | 0.0266 | 0.0146 | KLF4/TGFBR2/CCNB1 | 3 |
| BP | T cell differentiation | 0.0266 | 0.0146 | ERBB2/IRF1/TGFBR2 | 3 |
| BP | regulation of protein kinase B signaling | 0.0275 | 0.0151 | ERBB2/KLF4/NTRK2 | 3 |
| BP | cellular response to toxic substance | 0.0281 | 0.0154 | FOXO1/KLF4/TXNRD1 | 3 |
| BP | regulation of cholesterol metabolic process | 0.0285 | 0.0156 | ACACA/DGKQ | 2 |
| BP | regulation of muscle system process | 0.0314 | 0.0172 | FOXO1/KCNJ2/KLF4 | 3 |
| BP | cardiac muscle cell proliferation | 0.0314 | 0.0172 | TGFBR2/CCNB1 | 2 |
| BP | protein kinase B signaling | 0.0335 | 0.0183 | ERBB2/KLF4/NTRK2 | 3 |
| BP | positive regulation of growth | 0.0335 | 0.0183 | ERBB2/TGFBR2/CCNB1 | 3 |
| BP | lymphocyte proliferation | 0.0335 | 0.0183 | ERBB2/IRF1/TGFBR2 | 3 |
| BP | regulation of leukocyte differentiation | 0.0335 | 0.0183 | ERBB2/IRF1/TGFBR2 | 3 |
| BP | connective tissue development | 0.0335 | 0.0183 | ATF2/PKD1/TGFBR2 | 3 |
| BP | cell-cell adhesion via plasma-membrane adhesion molecules | 0.0335 | 0.0183 | KLF4/PKD1/TGFBR2 | 3 |
| BP | mononuclear cell proliferation | 0.0335 | 0.0183 | ERBB2/IRF1/TGFBR2 | 3 |
| BP | G1/S transition of mitotic cell cycle | 0.0345 | 0.0189 | KLF4/PKD1/CCNB1 | 3 |
| BP | mesoderm formation | 0.0345 | 0.0189 | KLF4/TXNRD1 | 2 |
| BP | peripheral nervous system development | 0.0345 | 0.0189 | ERBB2/NTRK2 | 2 |
| BP | regulation of oxidative stress-induced cell death | 0.0345 | 0.0189 | LANCL1/MCL1 | 2 |
| BP | reactive oxygen species metabolic process | 0.0348 | 0.019 | FOXO1/KLF4/TGFBR2 | 3 |
| BP | mesoderm morphogenesis | 0.0348 | 0.019 | KLF4/TXNRD1 | 2 |
| BP | negative regulation of cell adhesion | 0.0348 | 0.019 | ERBB2/IRF1/KLF4 | 3 |
| BP | tissue regeneration | 0.0348 | 0.019 | TGFBR2/CCNB1 | 2 |
| BP | regulation of cardiac muscle tissue growth | 0.0348 | 0.019 | TGFBR2/CCNB1 | 2 |
| BP | regulation of epithelial cell migration | 0.0348 | 0.019 | KLF4/TGFBR2/VEGFC | 3 |
| BP | vasculogenesis | 0.0348 | 0.019 | NTRK2/TGFBR2 | 2 |
| BP | outflow tract morphogenesis | 0.0348 | 0.019 | ATF2/TGFBR2 | 2 |
| BP | cellular response to mechanical stimulus | 0.0348 | 0.019 | IRF1/KCNJ2 | 2 |
| BP | striated muscle cell proliferation | 0.0355 | 0.0195 | TGFBR2/CCNB1 | 2 |
| BP | regulation of alcohol biosynthetic process | 0.0355 | 0.0195 | ACACA/DGKQ | 2 |
| BP | cell cycle G1/S phase transition | 0.0355 | 0.0195 | KLF4/PKD1/CCNB1 | 3 |
| BP | leukocyte proliferation | 0.0355 | 0.0195 | ERBB2/IRF1/TGFBR2 | 3 |
| BP | regulation of heart growth | 0.0374 | 0.0205 | TGFBR2/CCNB1 | 2 |
| BP | response to fatty acid | 0.0388 | 0.0212 | ACACA/CCNB1 | 2 |
| BP | camera-type eye development | 0.0391 | 0.0214 | KLF4/NTRK2/TGFBR2 | 3 |
| BP | regulation of T cell activation | 0.0391 | 0.0214 | ERBB2/IRF1/TGFBR2 | 3 |
| BP | regulation of cellular response to oxidative stress | 0.0391 | 0.0214 | LANCL1/MCL1 | 2 |
| BP | regulation of neural precursor cell proliferation | 0.0391 | 0.0214 | FOXO1/VEGFC | 2 |
| BP | gluconeogenesis | 0.0392 | 0.0215 | DGKQ/FOXO1 | 2 |
| BP | cellular response to alcohol | 0.0392 | 0.0215 | ACACA/KLF4 | 2 |
| BP | odontogenesis of dentin-containing tooth | 0.0397 | 0.0218 | ATF2/FOXO1 | 2 |
| BP | hexose biosynthetic process | 0.0409 | 0.0224 | DGKQ/FOXO1 | 2 |
| BP | cell death in response to oxidative stress | 0.0409 | 0.0224 | LANCL1/MCL1 | 2 |
| BP | regulation of alpha-beta T cell activation | 0.0409 | 0.0224 | IRF1/TGFBR2 | 2 |
| BP | protein homotetramerization | 0.0409 | 0.0224 | ACACA/KCNJ2 | 2 |
| BP | regulation of steroid biosynthetic process | 0.0409 | 0.0224 | ACACA/DGKQ | 2 |
| BP | activation of protein kinase activity | 0.0409 | 0.0224 | DGKQ/MAP3K11/TGFBR2 | 3 |
| BP | response to leukemia inhibitory factor | 0.0409 | 0.0224 | KLF4/VEGFC | 2 |
| BP | cellular response to leukemia inhibitory factor | 0.0409 | 0.0224 | KLF4/VEGFC | 2 |
| BP | mitochondrial ATP synthesis coupled electron transport | 0.0409 | 0.0224 | CCNB1/COX15 | 2 |
| BP | regulation of carbohydrate biosynthetic process | 0.0409 | 0.0224 | DGKQ/FOXO1 | 2 |
| BP | mitotic DNA damage checkpoint | 0.0409 | 0.0224 | ATF2/CCNB1 | 2 |
| BP | oligodendrocyte differentiation | 0.0409 | 0.0224 | ERBB2/NTRK2 | 2 |
| BP | regulation of response to oxidative stress | 0.0409 | 0.0224 | LANCL1/MCL1 | 2 |
| BP | negative regulation of mitotic cell cycle | 0.0409 | 0.0224 | ATF2/KLF4/CCNB1 | 3 |
| BP | ATP synthesis coupled electron transport | 0.0409 | 0.0224 | CCNB1/COX15 | 2 |
| BP | monosaccharide biosynthetic process | 0.0409 | 0.0224 | DGKQ/FOXO1 | 2 |
| BP | cellular response to external stimulus | 0.0409 | 0.0224 | FOXO1/IRF1/KCNJ2 | 3 |
| BP | cellular response to hydrogen peroxide | 0.0413 | 0.0226 | FOXO1/KLF4 | 2 |
| BP | regulation of cardiac muscle tissue development | 0.0418 | 0.0229 | TGFBR2/CCNB1 | 2 |
| BP | alpha-beta T cell differentiation | 0.0423 | 0.0232 | IRF1/TGFBR2 | 2 |
| BP | regulation of cyclin-dependent protein serine/threonine kinase activity | 0.0425 | 0.0233 | PKD1/CCNB1 | 2 |
| BP | positive regulation of reactive oxygen species metabolic process | 0.0425 | 0.0233 | KLF4/TGFBR2 | 2 |
| BP | negative regulation of leukocyte differentiation | 0.0427 | 0.0234 | ERBB2/IRF1 | 2 |
| BP | epithelial cell migration | 0.0427 | 0.0234 | KLF4/TGFBR2/VEGFC | 3 |
| BP | lymphocyte differentiation | 0.0431 | 0.0236 | ERBB2/IRF1/TGFBR2 | 3 |
| BP | epithelium migration | 0.0431 | 0.0236 | KLF4/TGFBR2/VEGFC | 3 |
| BP | cardiac muscle tissue growth | 0.0431 | 0.0236 | TGFBR2/CCNB1 | 2 |
| BP | mitotic spindle organization | 0.0431 | 0.0236 | PKD1/CCNB1 | 2 |
| BP | mitotic DNA integrity checkpoint | 0.0431 | 0.0236 | ATF2/CCNB1 | 2 |
| BP | regulation of cyclin-dependent protein kinase activity | 0.0431 | 0.0236 | PKD1/CCNB1 | 2 |
| BP | response to hypoxia | 0.0431 | 0.0236 | TGFBR2/VEGFC/CCNB1 | 3 |
| BP | tissue migration | 0.0431 | 0.0236 | KLF4/TGFBR2/VEGFC | 3 |
| BP | regulation of muscle adaptation | 0.0431 | 0.0236 | FOXO1/KLF4 | 2 |
| BP | eye development | 0.0434 | 0.0238 | KLF4/NTRK2/TGFBR2 | 3 |
| BP | peptidyl-tyrosine phosphorylation | 0.0434 | 0.0238 | DGKQ/ERBB2/NTRK2 | 3 |
| BP | peptidyl-tyrosine modification | 0.0439 | 0.024 | DGKQ/ERBB2/NTRK2 | 3 |
| BP | visual system development | 0.0439 | 0.024 | KLF4/NTRK2/TGFBR2 | 3 |
| BP | response to decreased oxygen levels | 0.0447 | 0.0245 | TGFBR2/VEGFC/CCNB1 | 3 |
| BP | sensory system development | 0.0447 | 0.0245 | KLF4/NTRK2/TGFBR2 | 3 |
| BP | negative regulation of T cell activation | 0.0447 | 0.0245 | ERBB2/IRF1 | 2 |
| BP | heart growth | 0.0447 | 0.0245 | TGFBR2/CCNB1 | 2 |
| BP | in utero embryonic development | 0.0448 | 0.0245 | PKD1/TGFBR2/CCNB1 | 3 |
| BP | regulation of organ growth | 0.0449 | 0.0246 | TGFBR2/CCNB1 | 2 |
| BP | regulation of cytokine biosynthetic process | 0.0454 | 0.0249 | IRF1/KLF4 | 2 |
| BP | regulation of epithelial cell proliferation | 0.0456 | 0.025 | ATF2/ERBB2/VEGFC | 3 |
| BP | regulation of glucose metabolic process | 0.0468 | 0.0257 | DGKQ/FOXO1 | 2 |
| BP | respiratory electron transport chain | 0.0468 | 0.0257 | CCNB1/COX15 | 2 |
| BP | formation of primary germ layer | 0.0497 | 0.0272 | KLF4/TXNRD1 | 2 |
| MF | transmembrane receptor protein kinase activity | 0.0091 | 0.005 | ERBB2/NTRK2/TGFBR2 | 3 |
| MF | mitogen-activated protein kinase kinase kinase binding | 0.0101 | 0.0056 | MAP3K11/TGFBR2 | 2 |
| MF | growth factor binding | 0.0155 | 0.0086 | ERBB2/NTRK2/TGFBR2 | 3 |
| MF | transcription cofactor binding | 0.0295 | 0.0163 | FOXO1/KLF4 | 2 |
| MF | kinase regulator activity | 0.031 | 0.0171 | DGKQ/KLF4/CCNB1 | 3 |
| MF | transmembrane receptor protein tyrosine kinase activity | 0.0407 | 0.0225 | ERBB2/NTRK2 | 2 |
| MF | protein kinase activator activity | 0.0465 | 0.0257 | DGKQ/CCNB1 | 2 |
| MF | beta-catenin binding | 0.0465 | 0.0257 | FOXO1/KLF4 | 2 |
| MF | activating transcription factor binding | 0.0465 | 0.0257 | ATF2/DGKQ | 2 |
| MF | kinase activator activity | 0.0465 | 0.0257 | DGKQ/CCNB1 | 2 |

**Supplementary Table S7.** Detailed KEGG descriptions and related OS gene names.

| **KEGG** | **Description** | **Pvalue** | **Qvalue** | **Gene Names** | **Count** |
| --- | --- | --- | --- | --- | --- |
| hsa04010 | MAPK signaling pathway | 0.0013 | 0.0011 | ATF2/ERBB2/MAP3K11/NTRK2/TGFBR2/VEGFC | 6 |
| hsa04151 | PI3K-Akt signaling pathway | 0.0225 | 0.019 | ATF2/ERBB2/MCL1/NTRK2/VEGFC | 5 |
| hsa04933 | AGE-RAGE signaling pathway in diabetic complications | 0.0247 | 0.0209 | FOXO1/TGFBR2/VEGFC | 3 |
| hsa04922 | Glucagon signaling pathway | 0.0247 | 0.0209 | ACACA/ATF2/FOXO1 | 3 |
| hsa04668 | TNF signaling pathway | 0.0247 | 0.0209 | ATF2/IRF1/VEGFC | 3 |
| hsa04926 | Relaxin signaling pathway | 0.0277 | 0.0234 | ATF2/TGFBR2/VEGFC | 3 |
| hsa04068 | FoxO signaling pathway | 0.0277 | 0.0234 | FOXO1/TGFBR2/CCNB1 | 3 |
| hsa04218 | Cellular senescence | 0.0399 | 0.0337 | FOXO1/TGFBR2/CCNB1 | 3 |

**Supplementary Table S8**. Parameters and results for screening the key lncRNA (ceRNA), which competes with **CCNB1** to bind miR-212-3p.

|  | **Seed** | **Results** |
| --- | --- | --- |
| SVM | 25 | **AC079834** |
| LASSO | 25 | **AC079834**, AP003027, BX005214, MDS2 |

Minimum lambda for LASSO =0.005. Venn result for SVM (Figure 6B) and LASSO (Figure 6C): **AC079834**. lncRNA, **AC079834,** selected in SVM (Figure 6D) and LASSO (Figure 6E) were used for axis (CCNB1–miR-212-3p–AC079834). The binding sites are listed in Supplementary Table S11 and Supplementary Figure 1.

**Supplementary Table S9**. Parameters and results for screening the key lncRNA (ceRNA), which competes with **PKD1** to bind miR-20b-5p.

|  | **Seed** | **Results** |
| --- | --- | --- |
| SVM | 25 | **AP000797** |
| LASSO | 25 | **AP000797**, AC073626, LINC00452, NKX2-1-AS1 |

Minimum lambda for LASSO = 0.005. Venn result for SVM and LASSO：**AP000797**. lncRNA, **AP000797,** selected in SVM (Figure 6D) and LASSO (Figure 6E) were used for axis (PKD1–miR-20b-5p–AP000797). The binding sites are listed in Supplementary Table S11 and Supplementary Figure 1.

**Supplementary Table S10**. The numerical results of ROC analysis.

|  | **Training Cohort** | | | **Validation Cohort** | | |
| --- | --- | --- | --- | --- | --- | --- |
|  | CCNB1–PKD1 pair | CCNB1 | PKD1 | CCNB1–PKD1 pair | CCNB1 | PKD1 |
| AUC[95%CI] | 0.828[0.660,0.935] | 0.733[0.554,0.870] | 0.793[0.620,0.912] | 0.917[0.658,0.996] | 0.528[0.261,0.783] | 0.889[0.623,0.990] |
| Sensitivity(%) | 66.67 | 66.67 | 53.33 | 100.00 | 100.00 | 100.00 |
| Specificity(%) | 100 | 84.21 | 100 | 83.33 | 25.00 | 75.00 |
| *p*-values | <0.001 | 0.011 | <0.001 | <0.001 | 0.901 | <0.001 |

**Abbreviation:** 95%CI, 95% confidence interval; *p*-values, significance level p (area = 0.5).

**Supplementary Table S11**. Binding site in 3′UTR of CCNB1 and PKD1.

| **Target** | **CCNB1** | **PKD1** |
| --- | --- | --- |
| miRNA | hsa-miR-212-3p | hsa-miR-20b-5p |
| miR Sequence | UAACAGUCUCCAGUCACGGCC | CAAAGUGCUCAUAGUGCAGGUAG |
| 3′ UTR | >CCNB1\|NM_031966\|3'UTR  1 cttgtaaacttgagttggagtactatatttacaaataaaattggcaccatgtgc**catctgtacatattactgttg**cattt  81 acttttaataaagcttgtggccccttttacttttttatagcttaactaatttgaa**tgtggttacttcctactgtag**ggta  161 gcggaaaagttgtcttaaaaggtatggtggggatatttttaaaaactccttttggtttacctggggatccaattgatgta  241 tatgtttatatactgggttcttgttttatatacctggcttttactttattaatatgagttactgaaggtgatggaggtat  321 ttgaaaattttacttccataggacatactgcatgtaagccaagtcatggagaatctgctgcatagctctattttaaagta  401 aaagtctaccaccgaatccctagtccccctgttttc**tgtttcttcttgtgattgctg**ccataattctaagttatttactt  481 ttaccactatttaagttatcaactttagctagtatcttcaaactttcactttgaaaaatgagaattttatattctaagcc  561  agttttcattttggttttgtgttttggttaataaaacaatactcaaatacaaaaaaaaaaaa | >PKD1\|NM_000296\|3'UTR  1 tcctccttcc tggcgggggt gggccgtgga gtcggagtgg acaccgctca gtattacttt  61 ctgccgctgt caaggccgag ggccaggcag aatggctgca cgtaggttcc ccagagagca  121 ggcaggggca tctgtctgtc tgtgggcttc a**gcacttta**a agaggctgtg tggccaacca  181 ggacccaggg tcccctcccc agctcccttg ggaaggacac agcagtattg gacggtttct  241 agcctctgag atgctaattt atttccccga gtcctcaggt acagcgggct gtgcccggcc  301 ccaccccctg ggcagatgtc ccccactgct aaggctgctg gcttcaggga gggttagcct  361 gcaccgccgc caccctgccc ctaagttatt acctctccag ttcctaccgt actccctgca  421 ccgtctcact gtgtgtctcg tgtcagtaat ttatatggtg ttaaaatgtg tatatttttg  481 tatgtcacta ttttcactag ggctgagggg cctgcgccca gagctggcct cccccaacac  541 ctgctgcgct tggtaggtgt ggtggcgtta tggcagcccg gctgctgctt ggatgcgagc  601 ttggccttgg gccggtgctg ggggcacagc tgtctgccag gcactctcat caccccagag  661 gccttgtcat cctcccttgc cccaggccag gtagcaagag agcagcgccc aggcctgctg  721 gcatcaggtc tgggcaagta gcaggactag gcatgtcaga ggaccccagg gtggttagag  781 gaaaagactc ctcctggggg ctggctccca gggtggagga aggtgactgt gtgtgtgtgt  841 gtgtgcgcgc gcgcacgcgc gagtgtgctg tatggcccag gcagcctcaa ggccctcgga  901 gctggctgtg cctgcttctg tgtaccactt ctgtgggcat ggccgcttct agagcctcga  961 caccccccca acccccgcac caagcagaca aagtcaataa aagagctgtc tgactgc |
| Score | 101–120 | 80 |
| Ref | <https://mirtarbase.cuhk.edu.cn/~miRTarBase/miRTarBase_2022/php/detail.php?mirtid=MIRT024969> | <http://mirdb.org/cgi-bin/target_detail.cgi?targetID=1977282> |
