# Supplementary figures and images for: An Oxidative Stress-Related Gene Pair (CCNB1/PKD1), Competitive Endogenous RNAs, and Immune-Infiltration Patterns Potentially Regulate Intervertebral Disc Degeneration Development
Source: Front Immunol. 2021 Nov 9;12:765382. doi: 10.3389/fimmu.2021.765382 (PMC8630707; doi:10.3389/fimmu.2021.765382)

Supplementary Figure


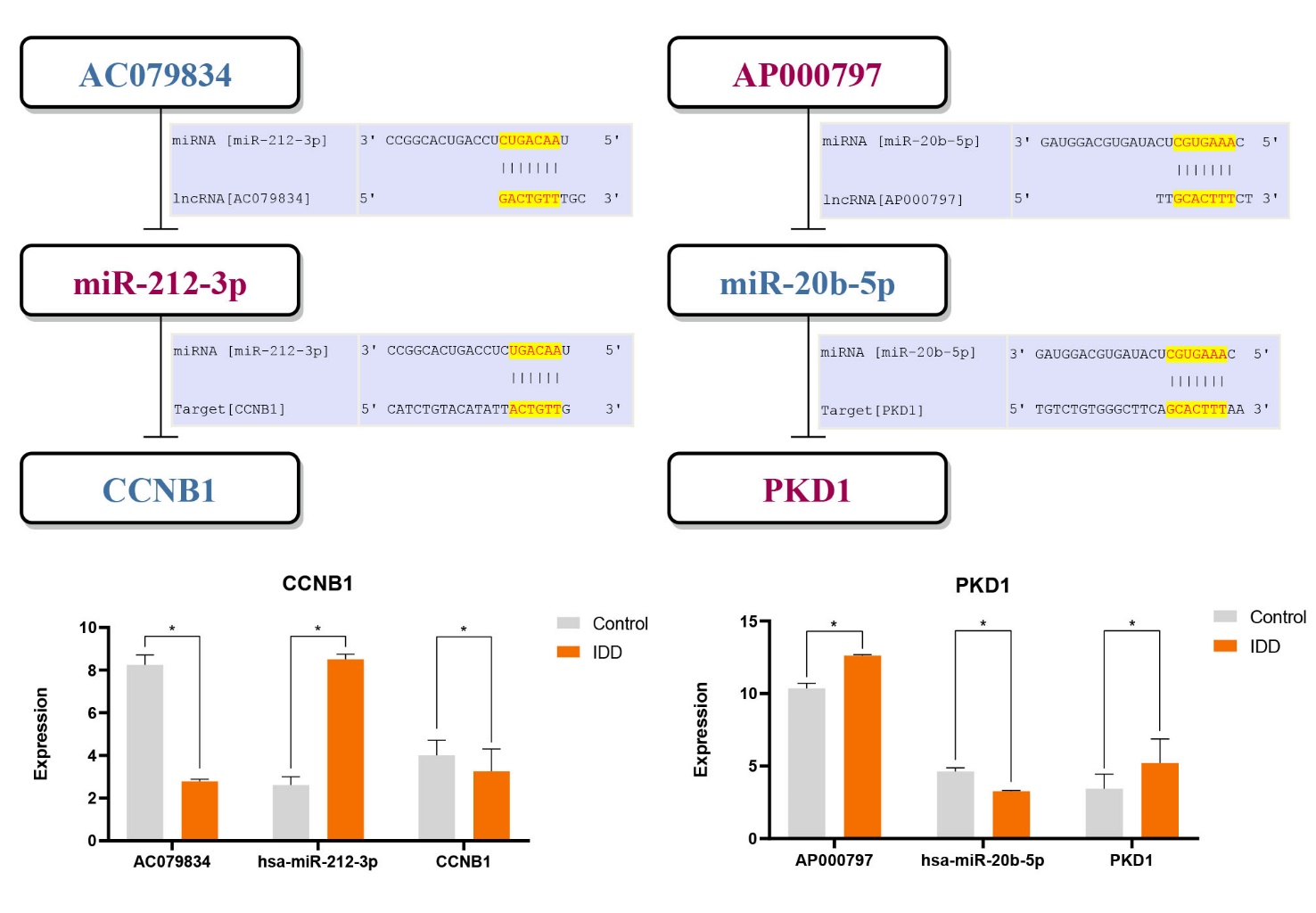


**Supplementary Figure 1. Binding sites for each axis.**

Supplement: Supplementary file 2 [file DataSheet_2.docx]
